# Conversion of placental hemogenic endothelial cells to hematopoietic stem and progenitor cells

**DOI:** 10.1038/s41421-024-00760-2

**Published:** 2025-01-28

**Authors:** Guixian Liang, Shicheng Liu, Chunyu Zhou, Mengyao Liu, Yifan Zhang, Dongyuan Ma, Lu Wang, Jing-Dong J. Han, Feng Liu

**Affiliations:** 1 Key Laboratory of Organ Regeneration and Reconstruction, State Key Laboratory of Membrane Biology, Institute of Zoology, Chinese Academy of Sciences, Beijing, China; 2 Institute for Stem Cell and Regeneration, Chinese Academy of Sciences, Beijing, China; 3 Beijing Institute for Stem Cell and Regenerative Medicine, Beijing, China; 4 University of Chinese Academy of Sciences, Beijing, China; 5 Peking-Tsinghua Center for Life Sciences, Academy for Advanced Interdisciplinary Studies, Center for Quantitative Biology (CQB), Peking University, Beijing, China; 6 State Key Laboratory of Experimental Hematology, National Clinical Research Center for Blood Diseases, Haihe Laboratory of Cell Ecosystem, Institute of Hematology and Blood Diseases Hospital, Chinese Academy of Medical Sciences and Peking Union Medical College, Tianjin, China; 7 School of Life Sciences, Shandong University, Qingdao, Shandong China

**Keywords:** Haematopoietic stem cells, Haematopoietic stem cells

## Abstract

Hematopoietic stem and progenitor cells (HSPCs) are critical for the treatment of blood diseases in clinic. However, the limited source of HSPCs severely hinders their clinical application. In the embryo, hematopoietic stem cells (HSCs) arise from hemogenic endothelial (HE) cells lining the major arteries in vivo. In this work, by engineering vascular niche endothelial cells (VN-ECs), we generated functional HSPCs in vitro from ECs at various sites, including the aorta-gonad-mesonephros (AGM) region and the placenta. Firstly, we converted mouse embryonic HE cells from the AGM region (aHE) into induced HSPCs (iHSPCs), which have the abilities for multilineage differentiation and self-renewal. Mechanistically, we found that VN-ECs can promote the generation of iHSPCs via secretion of CX3CL1 and IL1A. Next, through VN-EC co-culture, we showed that placental HE (pHE) cells, a type of extra-embryonic HE cells, were successfully converted into iHSPCs (pHE-iHSPCs), which have multilineage differentiation capacity, but exhibit limited self-renewal ability. Furthermore, comparative transcriptome analysis of aHE-iHSPCs and pHE-iHSPCs showed that aHE-iHSPCs highly expressed HSC-specific and self-renewal-related genes. Moreover, experimental validation showed that retinoic acid (RA) treatment promoted the transformation of pHE cells into iHSPCs that have self-renewal ability. Collectively, our results suggested that pHE cells possess the potential to transform into self-renewing iHSPCs through RA treatment, which will facilitate the clinical application of placental endothelial cells in hematopoietic cell generation.

## Introduction

Hematopoietic stem and progenitor cells (HSPCs) have the abilities of self-renewal and multilineage differentiation to maintain the supply of all blood lineages. HSPC transplantation is a curative therapy for blood diseases, in which HSPCs are the functional units^1^. Many efforts are underway to engineer HSPCs with the ability of long-term self-renewal and differentiation. Previous studies have developed directed differentiation strategies to differentiate human embryonic stem cells (ESCs) into hematopoietic lineages with an outcome of primitive myeloid-erythroid progenies^2,3^. Several groups have generated hematopoietic cells from human pluripotent stem cells (hPSCs) through morphogen-directed differentiation or transcription factor (TF)-mediated phenotypic conversion strategies^4–7^. Meanwhile, human endothelial cells (ECs) and mouse adult ECs were converted to immunocompetent HSPCs through TF-mediated conversion strategies^8–10^. However, these technologies have limitations in their initial cell sources, and the translation from bench to bedside requires the development of clinically safe protocols.

During mammalian embryonic development, the earliest definitive hematopoietic stem cells (HSCs) arise from a type of specified ECs in the dorsal aorta, known as hemogenic endothelial (HE) cells, through the endothelial-to-hematopoietic transition (EHT)^11–13^. Although erythroid-myeloid progenitors (EMPs) can be produced from HE cells in both arterial and venous vasculature^13–15^, HSCs are mostly restricted to arterial vessels, suggesting that arterial specification of HE cells is an essential prerequisite for engineering functional HSCs^12,16^. In addition to the aorta-gonad-mesonephros (AGM) region that is an embryonic hematopoietic organ, the mammalian placenta has been reported as an extraembryonic hematopoietic organ during development^17–24^. Meanwhile, the mouse placenta has been proposed to contain arterial-specific HE cells^25^. Moreover, previous studies have reported that HE cells from the AGM region can be transformed to HSPCs when co-cultured with OP9-DL1 cells in vitro^26–30^. However, long-term transplantation results of these HSPCs show a gradual decrease in chimerism, implying that they are not true HSCs^31^. Thus, it raises the question of whether embryonic arterial HE cells can be programmed to functional HSCs in vitro. Meanwhile, whether extraembryonic HE cells can be programmed to functional HSCs in vitro remains unknown.

To date, the molecular features underlying HSC development at the population and single-cell levels, as well as the mechanism controlling their maintenance and self-renewal during the in vitro induction, remain largely unresolved. Retinoic acid (RA) is a signaling molecule derived from vitamin A to regulate cell proliferation and differentiation. Several studies have shown that activation of RA signaling affects HE specification^32–35^. In addition to its role in HE specification, RA is also thought to have strong effects on the maintenance of HSC function. In mice, the addition of RA to short- and long-term repopulating HSCs increased maintenance and self-renewal capacity, which is mediated by activation of the RARγ receptor^36,37^. Moreover, RA treatment retains the long-term self-renewal capability of dormant HSCs in vitro via reducing the ROS level, suggesting the potential application of RA in hematopoietic induction^38^.

Here, we employed an engineered vascular niche to convert mouse HE cells from both the AGM region (aHE) and placenta (pHE) into induced HSPCs (iHSPCs). pHE-derived iHSPCs (pHE-iHSPCs) exhibit long-term reconstitution ability, but the chimerism declines over time, which is different from iHSPCs transformed from HE cells in the AGM region. By comparing iHSPCs derived from AGM and placenta, we revealed that self-renewal-associated genes were insufficiently expressed in the pHE-iHSPCs. Furthermore, experimental validation revealed that RA promotes the transformation of pHE cells into RA-pHE-iHSPCs, which show improved self-renewal abilities. Thus, our results provide another new cellular source for the induction of HSPC generation in vitro.

## Results

### Conversion of embryonic aHE cells to aHE-iHSCs

Bone marrow (BM)-derived fibroblastic OP9-DL1 cells, when co-cultured with aHE cells, can transform the latter into HSPCs^26,28^. However, long-term transplantation of these HSPCs showed a gradual decrease in chimerism^31^. Human umbilical vein endothelial cells (HUVECs) transduced with the *E4ORF1* gene can form vascular niche endothelial cells (VN-ECs) to promote the reprogramming of adult mouse ECs into hematopoietic cells^8,9^. Nevertheless, OP9-DL1 cells cannot convert reprogrammed adult mouse ECs into HSPCs, suggesting the different signals provided by VN-ECs and OP9-DL1 cells^9^. It remains unclear whether aHE cells can be transformed into HSPCs with long-term transplantation ability and self-renewal capacity when co-cultured with VN-ECs. Therefore, we established VN-ECs to study this possibility. First of all, HUVECs were purified by fluorescence-activated cell sorting (FACS) using the surface markers CD31 and CD144 from human umbilical veins (Supplementary Fig. S1a). Subsequently, HUVECs were transduced with the *E4ORF1* gene to generate HUVEC-E4 cells. Flow cytometry analysis results showed that 53.7% of HUVECs were successfully transduced with *E4ORF1* as indicated by GFP expression, consistent with the microscopy imaging data (Supplementary Fig. S1b, c). Additionally, an equal number of HUVEC and HUVEC-E4 cells were collected by FACS and then subjected to quantitative polymerase chain reaction (qPCR) analysis. The results showed that the expression of the *E4ORF1* gene was increased in the HUVEC-E4 cells (Supplementary Fig. S1d). It has been reported that the expression of *E4ORF1* can improve the survival of primary ECs in serum/cytokine-free conditions^39^. To test whether E4ORF1 plays a role in the survival of primary ECs, we employed serum/cytokine-free systems^39^. The microscopy results revealed that the survival of primary ECs was observed only in the presence of *E4ORF1* expression (Supplementary Fig. S1e, f). Additionally, qPCR analysis showed that *FGF2*, FGF receptor 2 (*FGFR2*) and *MAPK3* were highly expressed in the HUVEC-E4 cells, consistent with a previous report (Supplementary Fig. S1g)^39^.

**Fig. 1:**
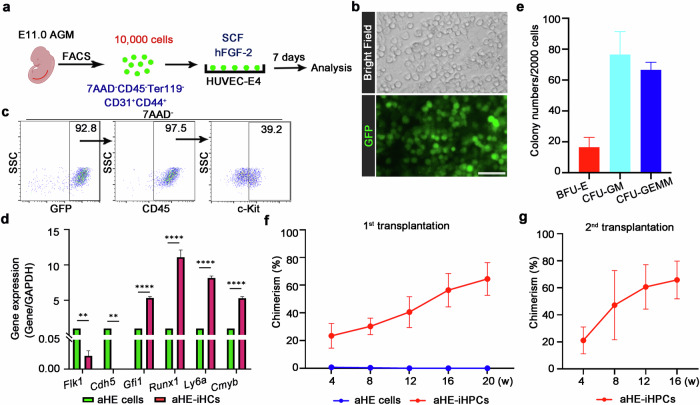
Conversion of embryonic aHE cells to aHE-iHSPCs. **a** Schematic illustration of hematopoietic induction from aHE cells at E11.0. 10,000 HE cells are sorted and directly seeded into 1 well of a 12-well plate that pre-coated with HUVEC-E4 cells. **b** Round hematopoietic cells generated from embryonic HE cells after 7 days of co-culture with HUVEC-E4ORF1. Scale bar, 50 μm. **c** Flow cytometry analysis showing the expression of CD45 and c-Kit after 7 days of co-culture with HUVEC-E4 cells. **d** qPCR analysis showing the downregulation of endothelial-related genes and upregulation of hematopoietic-related genes in aHE-iHCs generated by co-culturing aHE cells with HUVEC-E4 cells for 7 days. *n* = 3. **e** CFU-C assay of aHE-iHPCs (GFP^+^CD45^+^c-Kit^+^), *n* = 3. **f** The chimerism rates of primary recipients transplanted with aHE cells or aHE-iHPCs are evaluated at 4, 8, 12, 16 and 20 weeks, respectively. For aHE cell group, *n* = 3. For aHE-iHPC group, *n* = 4. **g** Donor chimerism of secondary recipients transplanted with aHE-iHPCs. *n* = 4. Error bars, means ± SD. **P < 0.01, ****P < 0.0001. *P*-values were calculated by two-tailed unpaired Student’s *t*-test.

Since placental tissues were used in the later part of this study, male B6-G/R mice were crossed with female C57BL/6 mice to distinguish cells with maternal and fetal origin in the placenta^25,40^. Therefore, cells in the AGM region and the fetal component of the placenta are GFP-positive (Supplementary Fig. S2a). To enrich embryonic HE cells, CD44, which has been identified as a marker of aHE cells, was used^41,42^. Immunofluorescence (IF) assay showed the expression of CD44, and flow cytometry results showed that CD44 marks 12.5% of ECs in the AGM region (Supplementary Fig. S2b, c). Then, aHE cells were purified by FACS and then co-cultured with HUVEC-E4 cells (Fig. 1a; Supplementary Fig. S2c). Freshly isolated aHE cells were unable to form hematopoietic colonies when cultured in the M3434 medium, indicating that aHE cell preparations were free of hematopoietic progenitor cell contamination (Supplementary Fig. S2d). After 7 days of co-culture, aHE cells committed to a hematopoietic fate, transitioning from CD45^–^ to CD45^+^ cells, acquiring hematopoietic morphology and expressing the corresponding markers (Fig. 1b, c). This indicates that aHE cells were transformed into aHE-induced hematopoietic cells (aHE-iHCs, GFP^+^CD45^+^). Meanwhile, qPCR analysis showed that the expression of hematopoietic genes, *Runx1*, *Ly6a*, *Gfi1* and *Cmyb*, was up-regulated in aHE-iHCs, whereas the expression of endothelial genes *Flk1* and *Cdh5* was down-regulated (Fig. 1d).

To investigate the phenotypic features of aHE-iHCs, flow cytometry analysis results showed that 39.2% of aHE-iHCs were featured by the HSC-associated marker c-Kit. These cells were named as aHE-induced hematopoietic progenitor cells (aHE-iHPCs) (Fig. 1c). To explore the hematopoietic properties of aHE-iHPCs, we performed colony-forming unit cell (CFU-C) assay, and the results showed that hematopoietic colonies, including burst-forming unit-erythoid (BFU-E), CFU-granulocyte-monocyte (CFU-GM) and CFU-mixed myeloid, megakaryocyte, and erythroid (CFU-GEMM) colonies were formed (Fig. 1e). To further determine the hematopoietic reconstitution ability of aHE-iHPCs in vivo, aHE-iHPCs (GFP^+^CD45^+^c-Kit^+^), together with adult BM helper cells (2 × 10^5^ nucleated cells), were transplanted into irradiated adult recipients. Peripheral blood of recipients was assessed for donor-derived chimerism at 4, 8, 12 and 16 weeks post-transplantation and BM of recipients was assessed for donor-derived chimerism at 20 weeks post-transplantation, respectively. We found that the aHE-iHPC population contained HSPCs (aHE-iHSPCs) that exhibited short-term (4 weeks) and long-term (20 weeks) hematopoietic reconstitution abilities (Fig. 1f; Supplementary Fig. S2e, f). Importantly, secondary transplantation demonstrated durable self-renewal activity, thus confirming the presence of functional HSCs within the aHE-iHPC population; and these functional HSCs were named aHE-iHSCs (Fig. 1g). Meanwhile, the transplantation of aHE cells did not yield short-term and long-term hematopoietic reconstitution, indicating that aHE cells were free of contaminated hematopoietic progenitor cells, consistent with the results of the colony formation assay (Fig. 1f; Supplementary Fig. S2d). Collectively, these results suggest that HUVEC-E4 cells can promote the transition of aHE cells into aHE-iHSCs, which have both differentiation and self-renewal capabilities.

### The regulatory mechanism of VN-ECs to promote aHE-iHSC formation

**Fig. 2:**
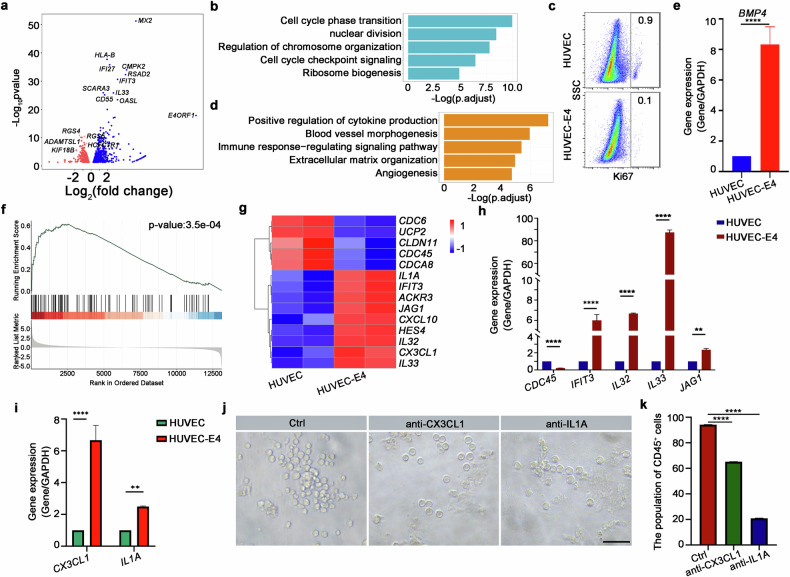
VN-ECs secreted cytokines to promote aHE-iHSC formation. **a** Volcano plot showing the DEGs for HUVEC and HUVEC-E4 populations. **b** GO terms enriched in HUVEC population. **c** Flow cytometry analysis showing the expression of Ki67 in HUVEC and HUVEC-E4 populations. **d** GO terms enriched in HUVEC-E4. **e** qPCR analysis showing the expression of *BMP4* in HUVEC and HUVEC-E4 populations, respectively. *n* = 3. **f** GSEA indicated that Toll-like receptor signaling was enriched in HUVEC-E4. **g** Heatmap showing the expression of genes associated with inflammatory response in HUVEC and HUVEC-E4 populations. **h** qPCR analysis showing the expression of *CDC45, IFIT3, IL32, IL33* and *JAG1* in HUVEC and HUVEC-E4 populations, respectively. *n* = 3. **i** qPCR analysis showing the expression of *CX3CL1* and *IL1A* in HUVEC and HUVEC-E4 populations. **j** Brightfield microscopy images of round hematopoietic cells generated from HE cells with the treatment of anti-CX3CL1 and anti-IL1A neutralizing antibodies, respectively. Scale bar, 50 μm. **k** Bar chart showing the proportion of CD45^+^ aHE-iHCs treated with anti-CX3CL1 and anti-IL1A neutralizing antibodies, respectively. *n* = 3. Error bars, means ± SD. **P < 0.01, ****P < 0.0001. *P*-values were calculated by two-tailed unpaired Student’s *t*-test.

To investigate the underlying molecular signals of VN-ECs in promoting aHE-iHSC formation, we performed bulk RNA sequencing (bulk RNA-seq) using sorted HUVEC and HUVEC-E4 populations (Supplementary Fig. S1c). As expected, *E4ORF1* gene was highly expressed in HUVEC-E4 cells (Fig. 2a). To investigate the enriched molecular signals, Gene Ontology (GO) term analysis was performed. The upregulated genes in HUVECs were enriched in the regulation of cell cycle and cell division, indicating that the expression of *E4ORF1* inhibits cell proliferation (Fig. 2b). Meanwhile, flow cytometry analysis showed that the proportion of Ki67-positive cells decreased in HUVEC-E4 cells, which was consistent with the results of transcriptomic analysis (Fig. 2c). Moreover, the up-regulated genes in HUVEC-E4 cells were enriched in the regulation of angiogenesis and blood vessel morphogenesis, consistent with the fact that E4ORF1 promotes the angiogenic functions of ECs (Fig. 2d)^39^. Furthermore, *BMP4*, which has been reported to promote EHT, was highly expressed in HUVEC-E4 cells (Fig. 2e)^9^. Gene Set Enrichment Analysis (GSEA) indicated that Toll-like receptor signaling was activated in HUVEC-E4 cells, which has been reported to promote HSC generation (Fig. 2f)^43–45^. Additionally, Notch signaling-related genes, including *JAG1* and *HES4*, which have been reported to play roles in EHT, were highly expressed in HUVEC-E4 cells, suggesting that HUVEC-E4 cells facilitate the EHT process and support HSC generation (Fig. 2g, h).

Furthermore, according to previous studies by us and others, inflammatory signaling is essential for HSC emergence^43,44,46,47^. GO analysis showed that terms related to cytokine production and immune response signaling pathway were enriched in HUVEC-E4 cells, suggesting that HUVEC-E4 cells may secrete cytokines to promote the conversion of HE cells to HSCs (Fig. 2d). IL-33, which recently has been reported to play roles in HE transition states, was highly expressed in HUVEC-E4 cells (Fig. 2a, g, h)^48,49^. Additionally, transcriptomic analysis showed that genes associated with inflammatory response, including *CX3CL1* and *IL1A*, were highly expressed in HUVEC-E4 cells (Fig. 2g, i). To investigate whether CX3CL1 is required to promote HE cell transition in vitro, a neutralizing antibody of CX3CL1 was used. Although the anti-CX3CL1 antibody did not impair cell viability, the number of induced HSPCs was decreased when CX3CL1 was blocked, suggesting that CX3CL1 likely promotes HE cell transition in vitro (Fig. 2j, k). Furthermore, to investigate whether the pro-inflammatory cytokine, IL1A, plays a role in the EHT process in vitro, we employed an IL1A-neutralizing antibody and found that inhibition of IL1A attenuated the induction of HSPCs (Fig. 2j, k). Collectively, these results showed that VN-ECs can promote the transition of HE cells to iHSCs by secretion of cytokines, including CX3CL1 and IL1A. Together, these results indicated that the expression of *E4ORF1* in HUVEC-E4 cells can inhibit cell proliferation, promoting angiogenic function and hematopoietic cell generation.

### Conversion of pHE cells into pHE-iHSPCs

As mentioned above, HUVEC-E4 can promote the transition of aHE cells into aHE-iHSCs with long-term reconstituting abilities and self-renewal capabilities, we thus wonder whether extra-embryonic pHE cells, which contribute to HSPCs and macrophages in vivo, harbor the capacity to generate HSPCs in vitro^17–19,25,50^. To this end, GFP-positive placental cells were purified by FACS to eliminate the contamination of maternal cells (Supplementary Figs. S2a, S3a). IF assay showed the expression of CD44 in placental ECs, and flow cytometry analysis showed that CD44 labeled 9.6% (3.2/(3.2 + 30.2) × 100%) of placental ECs (Supplementary Fig. S3a, b). Meanwhile, freshly isolated pHE cells cultured in the M3434 medium led to no colony formation, indicating that pHE cell preparations were free of any contaminated hematopoietic progenitor cells (Supplementary Fig. S3c).

**Fig. 3:**
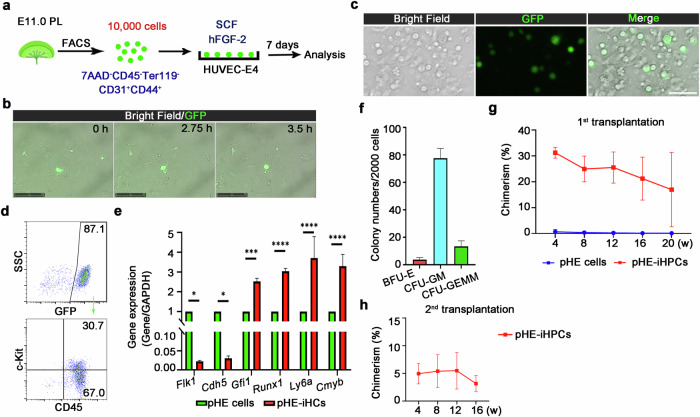
Conversion of pHE cells to pHE-iHSPCs. **a** Schematic illustration of hematopoietic induction from pHE cells at E11.0. 10,000 HE cells are sorted and directly seeded into 1 well of a 12-well plate that pre-coated with HUVEC-E4 cells. **b** Imaging of morphological change of pHE cells co-cultured on HUVEC-E4 cells. Scale bars, 125 μm. **c** GFP-positive round hematopoietic cells generated from pHE cells after 7-day co-culture with HUVEC-E4 cells. Scale bar, 50 μm. **d** Flow cytometry analysis showing the expression of CD45 and c-Kit after co-culturing pHE cells with HUVEC-E4 cells for 7 days. **e** qPCR analysis showing the downregulation of endothelial-related genes and upregulation of hematopoietic-related genes in pHE-iHCs generated by co-culturing pHE cells with HUVEC-E4 cells for 7 days. *n* = 3. **f** CFU-C assay of FACS-sorted 2000 pHE-iHPCs (GFP^+^CD45^+^c-Kit^+^). *n* = 3. **g** The chimerism rates of primary recipients transplanted with pHE cells or pHE-iHPCs were evaluated at 4, 8, 12, 16 and 20 weeks, respectively. For the pHE cell group, *n* = 3. For the pHE-iHPC group, *n* = 5. **h** Donor chimerism of secondary recipients transplanted with pHE-iHPCs. *n* = 5. Error bars, means ± SD. *P < 0.05, ***P < 0.001, ****P < 0.0001. *P*-values were calculated by two-tailed unpaired Student’s *t*-test.

To initiate conversion, pHE cells were purified by FACS and then co-cultured with HUVEC-E4 cells (Fig. 3a). Time-lapse imaging showed the dynamic transition of pHE cells from a flattened to a rounded morphology (Fig. 3b; Supplementary Video S1). After 7 days of co-culture, pHE cells committed to a hematopoietic fate, transitioning from CD45^–^ to CD45^+^ cells, and acquiring hematopoietic morphology and corresponding markers (Fig. 3c, d). Meanwhile, qPCR analysis showed decreased expression of endothelial-related genes and increased expression of hematopoietic-related genes in FACS-sorted pHE-iHCs (GFP^+^CD45^+^) when compared to pHE cells (Fig. 3e). To investigate the phenotypic markers in the pHE-iHCs, flow cytometry analysis showed that the HSC-associated marker c-Kit was expressed (Fig. 3d). To explore the hematopoietic properties of pHE-iHPCs (GFP^+^CD45^+^c-Kit^+^), CFU-C assay was performed to show the formation of BFU-E, CFU-GM and CFU-GEMM colonies (Fig. 3f). Furthermore, to investigate the hematopoietic function of pHE-iHPCs, transplantation analysis was performed, and the results showed that the pHE-iHPC population contained HSPCs (pHE-iHSPCs), which exhibited short-term and long-term hematopoietic reconstitution abilities, suggesting that pHE cells have the potential to transform into HSPCs in vitro (Fig. 3g; Supplementary Fig. S3d, e). Similar to aHE cells, transplantation of pHE cells also did not yield short-term or long-term hematopoietic reconstitution, consistent with the results of the colony formation assay (Fig. 3g). However, when directly comparing the hematopoietic output functions of aHE cells and pHE cells, the transplantation results showed an increase in chimerism over time in the aHE-iHPC group, whereas the pHE-iHPC group exhibited a decline over time, implying that pHE-iHPCs are different from aHE-iHPCs (Figs. 1f, 3g). Furthermore, the secondary transplantation experiment showed that pHE-iHPCs engrafted at low levels in lethally irradiated mice (Fig. 3h). Collectively, these results suggest that HUVEC-E4 cells can promote the transition of pHE cells into iHSPCs with long-term differentiation ability; however, the self-renewal ability of pHE-iHSPC is limited.

### Single-cell transcriptome reveals different cell types induced from aHE cells and pHE cells

**Fig. 4:**
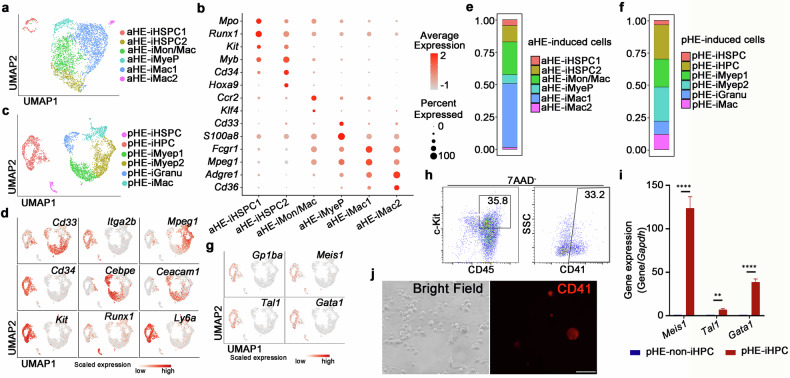
Single-cell transcriptomics identify diverse hematopoietic populations induced by aHE and pHE cells. **a** UMAP visualization of scRNA-seq data from aHE-iHCs. **b** Dot plots showing landmark genes in each cluster from aHE-iHCs. **c** UMAP visualization of scRNA-seq data from pHE-iHCs. **d** Feature plots showing maker genes in each cluster from pHE-iHCs. **e**, **f** The fraction of cell types in the aHE-iHCs (**e**) and pHE-iHCs (**f**). **g** Feature plots showing the expression of megakaryocyte-associated genes in the pHE-iHCs. **h** Flow cytometry analysis showing the expression of CD45, c-Kit and CD41 in the pHE-iHCs. **i** qPCR analysis showing the expression of *Meisi, Tal1* and *Gata1* in FACS-sorted pHE-derived non-iHPCs (pHE-non-iHPCs) and pHE-iHPCs, respectively. Error bars, means ± SD. **P < 0.01, ****P < 0.0001. *P*-values were calculated by two-tailed unpaired Student’s *t*-test. **j** Microscopy images showing the expression of CD41 in large cells in the pHE-iHCs. Scale bar, 50 μm.

To investigate the different hematopoietic outputs of aHE cells and pHE cells, we performed 10X Genomics-based single-cell RNA sequencing (scRNA-seq) on the GFP^+^CD45^+^ cells after co-culturing aHE and pHE cells with HUVEC-E4 cells for 7 days to get an unbiased perspective of the induced cells (Supplementary Fig. S4a). For scRNA-seq of aHE-iHCs, 3012 cells passed the stringent quality control and were retained for further analysis. We employed Uniform Manifold Approximation and Projection (UMAP) analysis to annotate cell types and identified six cell types based on differently expressed genes (DEGs), including aHE-iHSPC1, which was identified by the expression of *Mpo*, *Runx1* and *Kit*; aHE-iHSPC2, which was characterized by the expression of *Myb*, *Cd34* and *Hoxa9*; aHE-induced monocyte/macrophage (aHE-iMon/Mac) that highly expresses *Ccr2*; aHE-induced myeloid progenitor (aHE-iMyeP), which was characterized by the expression of *Cd33*; and two subpopulations of aHE-induced macrophage (aHE-iMac), which were identified by the expression of *Mpeg1* and *Adgre1* (Fig. 4a, b; Supplementary Fig. S4b). For scRNA-seq of pHE-iHCs, 2966 cells passed the stringent quality control and were retained for further analysis. We identified six cell types based on DEGs, including pHE-iHSPC, which was characterized by the expression of *Runx1*; pHE-iHPC was identified by the expression of *Kit*, *Itga2b, Ly6a* and *Cd34*; pHE-iMyeP, including two subclusters, were identified by the expression of *Cd33 and Cebpe*; pHE-induced granulocyte (pHE-iGranu) was characterized by the expression of *Ceacam1*; pHE-iMac was characterized by the expression of *Mpeg1* (Fig. 4c, d; Supplementary Fig. S4c).

Furthermore, to integrate the hematopoietic outputs of aHE cells and pHE cells, UMAP analysis revealed that the hematopoietic outputs of aHE cells and pHE cells were mostly divided into two distinct regions (Supplementary Fig. S4d). To illustrate the abundance of cell types, cell proportion analysis showed that > 50% of aHE-iHCs are monocytes and/or macrophages, while in pHE-iHCs, the most abundant cells are myeloid progenitors (Fig. 4e, f). Importantly, we found that pHE cells give rise to iHPC, which specifically expressed megakaryocyte-associated genes, including *Gp1ba*, *Meis1*, *Tal1* and *Gata1*, implying that iHPC may contain megakaryocyte-related phenotypes (Fig. 4g). To explore the phenotypic features of pHE-iHPC, flow cytometry analysis showed that 11.9% (35.8% × 33.2%) of pHE-induced cells highly express *Itga2b*, *Cd45* and *Kit* (Fig. 4h). Giemsa staining of FACS-sorted pHE-iHPCs (CD45^+^CD41^+^c-Kit^+^) showed the representative morphology of these cells (Supplementary Fig. S4e). Moreover, qPCR analysis of FACS-sorted pHE-non-iHPCs (CD45^+^CD41^–^c-Kit^+^) and pHE-iHPCs showed high expression of *Meis1*, *Tal1* and *Gata1* in the pHE-iHPCs (Fig. 4i). Additionally, when compared the hematopoietic morphology of aHE-iHCs and pHE-iHCs, the results showed that there are larger-size round cells in the group pf pHE-iHCs, and the fluorescence staining results showed that these cells are CD41-positive (Fig. 4j; Supplementary Fig. S4f). In summary, these results showed that though both co-cultured with HUVEC-E4 cells, aHE cells and pHE cells gave rise to different hematopoietic output, aHE cells generated more mono-macrophages, while pHE cells contributed to more myeloid progenitors and iHPCs with megakaryocyte-associated phenotype.

### Transcriptome comparison reveals the insufficient expression of self-renewal genes in the pHE-iHSPCs

**Fig. 5:**
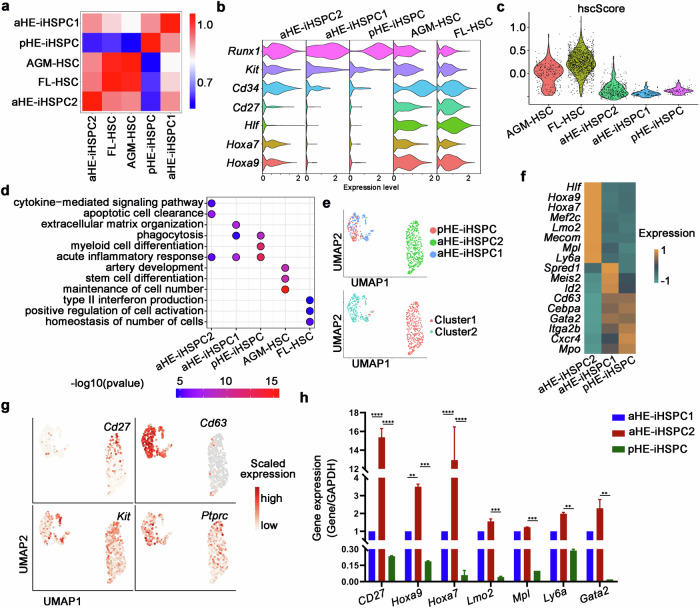
Transcriptomic analysis of aHE-iHSPCs and pHE-iHSPCs. **a** UMAP visualization of iHSPC clusters with AGM-HSC and FL-HSC. **b** Violin plot showing the log-normalized expression of representative HSC-related genes in iHSPC clusters, AGM-HSC and FL-HSC. **c** Violin plot showing the different hscScore values among iHSPC clusters, AGM-HSC and FL-HSC. **d** Enriched GO terms for biological processes in the five groups. *P*-value was calculated using hypergeometric test and adjusted for multiple testing using Benjamini-Hochberg correction. **e** UMAP visualization of pHE-iHSPCs and aHE-iHSPCs (upper). Lower, Cluster 1 mainly comprises aHE-iHSPC2, while Cluster 2 comprises pHE-iHSPCs and aHE-iHSPC1. **f** Heatmap showing the expression of HSC self-renewal-related genes and other hematopoietic-related genes in pHE-iHSPC, aHE-iHSPC1 and aHE-iHSPC2. **g** UMAP showing the expression of HSC self-renewal-related genes and other hematopoietic-related genes in pHE-iHSPCs, aHE-iHSPC1 and aHE-iHSPC2. **h** qPCR analysis showing the expression of HSC self-renewal-related genes in aHE-iHSPCs and pHE-iHSPCs, respectively. *n* = 3. Error bars, means ± SD. **P < 0.01, ***P < 0.001, ****P < 0.0001. *P*-values were calculated by two-tailed unpaired Student’s *t*-test.

To determine whether induced cells contain HSCs, we employed the score of HSC signature (hscScore), which has been reported^51^. The results showed that aHE-iHSPC1, aHE-iHSPC2 and pHE-iHSPC have higher scores compared with other clusters, indicating that they match transcriptional features of HSC (Supplementary Fig. S5a, b). To characterize the transcriptional features of iHSPC populations, we integrated our iHSPC clusters with a published scRNA-seq dataset of the HSCs from the AGM and fetal liver (FL) (Fig. 5a; Supplementary Fig. S5c–e)^52^. The integrated transcriptomic analysis showed that the aHE-iHSPC2 is highly correlated with HSCs from FL (Fig. 5a). Similar to FL-HSC, aHE-iHSPC2 highly expresses *Kit*, *Runx1*, *Hoxa7* and *Hoxa9* (Fig. 5b). Meanwhile, hscScore analysis also suggested that FL-HSC has a higher score than AGM-HSCs (Fig. 5c). However, although transcriptome analysis showed that aHE-iHSPC2 is similar to FL-HSCs, hscScore analysis showed that FL-HSCs has a higher score than aHE-iHSPC2, indicating that iHSPCs both derived from aHE and pHE cells, are different from HSCs in vivo (Fig. 5c). Additionally, GO analysis showed that the term related to artery development is enriched in AGM-HSC, consistent with the development of AGM-HSC in vivo (Fig. 5d)^11,12^. The term related to interferon production is enriched in FL-HSC, in agreement with that interferon signaling plays roles in HSPC expansion (Fig. 5d)^53,54^. In addition, the term of acute inflammatory response is enriched in aHE-iHSPC1, aHE-iHSPC2 and pHE-iHSPC, suggesting that inflammatory signaling genes are highly expressed in hematopoietic induction in vitro (Fig. 5d)^44,46,55^.

Furthermore, to explore the transcriptomic features of the iHSPC population, aHE-iHSPC1, aHE-iHSPC2 and pHE-iHSPC were integrated for further analysis. UMAP analysis showed that aHE-iHSPC1 and pHE-iHSPC mixed together, while aHE-iHSPC2 was separated from the others (Fig. 5e). To analyze the DEGs of aHE-iHSPC1, aHE-iHSPC2 and pHE-iHSPC, we found that HSC-specific marker *Hlf* and self-renewal-related genes *Hoxa9*, *Lmo2* and *Hoxa7*, are highly expressed in aHE-iHSPC2 (Fig. 5f). To enrich iHSPC populations, we examined surface markers of these populations. We found that CD27, which is highly expressed in lymphoid progenitors and had also been reported as an HSC marker, could be used to enrich aHE-iHSPC2 (Fig. 5g)^56^. In addition, CD63, which had been reported as an HSC marker recently, could be used to enrich aHE-iHSPC1 and pHE-iHSPC (Fig. 5f, g)^57^. Then we used CD27, CD45 and c-Kit to collect aHE-iHSPC2 (GFP^+^CD27^+^CD45^+^c-Kit^+^) and CD63, CD45 and c-Kit to collect aHE-iHSPC1 (GFP^+^CD63^+^CD45^+^c-Kit^+^) for further analysis (Supplementary Fig. S5f). Giemsa staining showed the progenitor morphology of aHE-iHSPC1 and aHE-iHSPC2, respectively (Supplementary Fig. S5f). To explore the percentage of iHPSCs produced by aHE or pHE cells, we calculated the number of iHSPCs after co-culturing HE cells with HUVEC-E4 cells for 7 days. By calculating the number of aHE-iHSPCs generated from aHE cells, we found that 10,000 aHE cells produced ~1624 ± 246 aHE-iHSPC1 cells and 15,404 ± 789 aHE-iHSPC2, respectively (Supplementary Fig. S5g). Additionally, we discovered that 10,000 pHE cells generated ~4010 ± 1071 pHE-iHSPCs (Supplementary Fig. S5g). qPCR analysis of FACS-sorted aHE-iHSPC1, aHE-iHSPC2 and pHE-iHSPC cells confirmed that self-renewal-related genes *Hoxa9*, *Lmo2* and *Hoxa7* are highly expressed in the aHE-iHSPC2 cells (Fig. 5h). Taken together, these results suggest that the transcriptome of pHE-iHSPC is similar to that of aHE-iHSPC1 but differs from that of aHE-iHSPC2, and that the expression of self-renewal-related genes is insufficient in pHE-iHSPC.

### RA treatment promotes the induction of self-renewing HSPCs from pHE cells

**Fig. 6:**
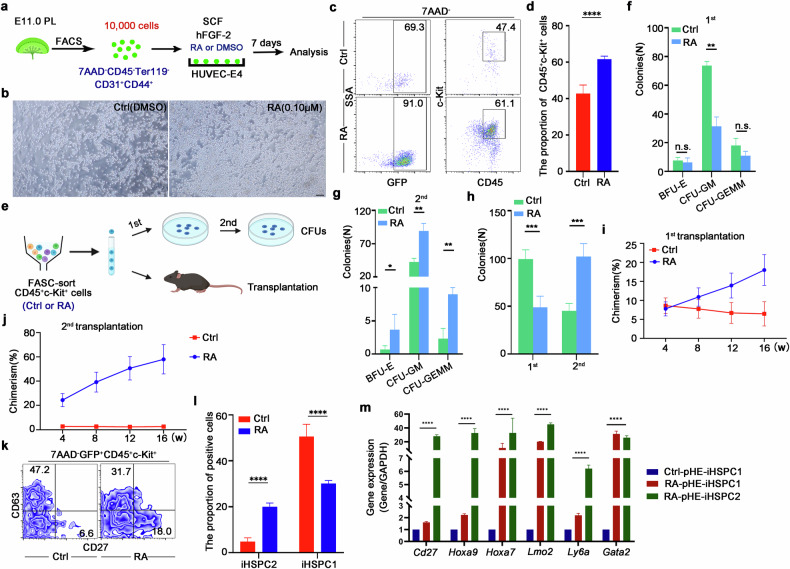
RA treatment improves the self-renewal ability of pHE-iHSPCs. **a** Model depicting the induction process of pHE cells with RA treatment (RA group) or without RA treatment (DMSO treatment, Ctrl). **b** Round hematopoietic cells generated from pHE cells after 7 day co-culture with HUVEC-E4 cells in Ctrl and RA groups. Scale bar, 50 μm. **c** Flow cytometry analysis showing the proportion of CD45^+^c-Kit^+^ cells in Ctrl and RA groups. **d** Bar plot indicating the percentage of CD45^+^c-Kit^+^ cells measured by FACS. Data are presented as means ± SD. *n* = 3. **e** Schematic illustration of serial CFU-C analysis and transplantation experiment of CD45^+^c-Kit^+^ cells. **f** The 1st CFU-C assay of 2000 FACS-sorted CD45^+^cKit^+^ iHPCs from Ctrl and RA groups, respectively. *n* = 3. **g** The 2nd CFU-C assay using an equal number of cells derived from the 1st colony formation assay of the Ctrl and RA groups, respectively. *n* = 3. **h** Total colonies are represented for the first and second plating. *n* = 3. **i** Donor chimerism of the 1st recipients transplanted with pHE-iHPCs from Ctrl and RA groups, respectively. *n* = 5. **j** Donor chimerism of the 2nd recipients transplanted with whole BM cells from the 1st recipients of Ctrl and RA groups, respectively. *n* = 5. **k** Flow cytometry analysis showing the proportion of CD27^+^ and CD63^+^ pHE-iHSPCs in Ctrl and RA groups. **l** Bar plot indicating the percentage of CD27^+^ iHSPC1 and CD63^+^ iHSPC2 in Ctrl and RA groups measured by FACS. **m** qPCR analysis of the expression of HSC self-renewal-related genes in Ctrl and RA groups, respectively. *n* = 3. Error bars, means ± SD. ***P < 0.001, ****P < 0.0001. *P-*values were calculated by two-tailed unpaired Student’s *t*-test.

To investigate whether the insufficient expression of self-renewal genes in the pHE-iHSPC had led to the low-level engraftment in lethally irradiated mice in the second transplantation assay, we focused on signaling pathways which can enhance the self-renewal ability of HSC. RA signaling had been reported to modulate HSC activity in the AGM region, long-term HSC mobilization in the BM and long-term repopulation activity of cultured HSCs in vitro; so we wondered whether RA signaling plays a role in pHE-iHSPC induction^32–37^. To this end, we added RA when co-culturing pHE cells with HUVEC-E4 cells (RA group). At the same time, DMSO was added to the control group (Ctrl group) (Fig. 6a). As expected, after 7 days of co-culture, pHE cells with RA or DMSO treatment both committed to a hematopoietic fate, transitioning from CD45^–^ cells to CD45^+^ cells, acquiring hematopoietic morphology and expressing corresponding genes (Fig. 6b, c). Meanwhile, microscopy imaging showed that the number of round cells was increased in the RA group (Fig. 6b). Flow cytometry analysis confirmed that the proportion of pHE-iHPCs also increased in the RA group (Fig. 6c, d). To explore the hematopoietic properties of pHE-iHPC after RA treatment, a serial CFU-C assay was performed as previously reported^38^. An equal number (2000 cells) of RA-pHE-iHPCs and Ctrl-pHE-iHPCs were sorted out to perform colony formation assays, respectively (Fig. 6e). The first CFU-C assay showed that a greater number of CFU-GM colonies were formed in the pHE-iHPC group, leading to a significant reduction in the total number of colonies in the RA-pHE-iHPC group (Fig. 6f, h). Based on our previous data, we propose that pHE cells give rise to a larger number of myeloid progenitors, which may contribute to an increased formation of colonies exhibiting granulocyte-macrophage features (Figs. 4f, 6f). Furthermore, the second CFU-C assay results indicated an increased number of colonies generated by RA-pHE-iHPCs, suggesting that RA-pHE-iHPCs contain HSCs possessing superior self-renewal capacity compared to those in pHE-iHPCs (Fig. 6g, h). Moreover, to explore the hematopoietic properties of pHE-iHPC after RA treatment, RA-pHE-iHPC and Ctrl-pHE-iHPC were collected and mixed with nucleated cells from BM and then transplanted into lethally irradiated mice. The results showed that both RA-pHE-iHPC and Ctrl-pHE-iHPC exhibited short-term and long-term hematopoietic reconstitution abilities (Fig. 6i). However, the chimerism of RA-pHE-iHPC increased over time, whereas the chimerism of Ctrl-pHE-iHPC decreased over time (Fig. 6i). To explore whether these cells had self-renewal ability, BM cells were collected and then injected into lethally irradiated mice. Second transplantation assay showed that BM cells from the RA-pHE-iHPC group could reconstruct the hematopoietic system, while those from the Ctrl-pHE-iHPC group failed, supporting that the RA-pHE-iHPC population contains HSCs with self-renewal ability, similar to aHE-iHSCs (Fig. 6j).

To investigate the proportion of iHSPCs with or without RA treatment, we used CD27 and CD63 to identify the different subtypes of iHSPCs. Flow cytometry analysis showed that the proportion of GFP^+^CD27^+^CD45^+^c-Kit^+^ cells (referred to as pHE-iHSPC2) in the RA group was higher than that in the Ctrl group, whereas the proportion of GFP^+^CD63^+^CD45^+^c-Kit^+^ cells (referred to as pHE-iHSPC1) in the RA group was lower than that in the Ctrl group, suggesting that RA promoted the generation of pHE-iHSPC2 (Fig. 6k, l). Given that the number of pHE-iHSPC2 generated in the Ctrl group was small and that it was difficult to collect sufficient Ctrl-pHE-iHSPC2 cells, we used Ctrl-pHE-iHSPC1 for further comparative analysis (Fig. 6k, l). To investigate the expression of genes associated with self-renewal, qPCR analysis was performed and the results showed that the expression levels of self-renewal-related genes, including *Hoxa9*, *Hoxa7* and *Lmo2*, were increased in RA-pHE-iHSPC2, when compared to Ctrl-pHE-iHSPC1; and this was consistent with the comparison results of aHE-iHPSC2 and pHE-iHSPC (Fig. 6m). Collectively, these results revealed that RA promoted the induction of pHE-iHSPC2, which highly expressed self-renewal-related genes. In summary, these results suggested that RA signaling can promote the formation of pHE-iHSPC and improve their self-renewal ability.

## Discussion

In this study, we employed engineered VN-ECs to convert mouse aHE cells into iHSPCs and revealed that aHE-iHSPCs have long-term reconstitution and self-renewal capacity, similar to that of authentic HSCs in vivo. Moreover, we found that pHE cells could be successfully converted into iHSPCs with long-term differentiation capacity but limited self-renewal ability. Comparative analysis of aHE-iHSPC subpopulations and pHE-iHSPC showed that self-renewal-associated genes are specifically expressed in aHE-iHSPC2, and functional experimental validation revealed that RA promotes the transformation of pHE into pHE-iHSPC that has self-renewal ability.

Several studies have described the presence of HE cells in different anatomic sites during embryonic development^25,42^. In previous reports, HE cells in the yolk sac gave rise to EMPs that have no lymphoid potential^58^, and HE cells in the AGM region developed into HSCs^12^, while in our previous work, we identified that HE cells in the placenta gave rise to macrophages^25^. Previous studies showed that the activation of the arterial program is essential for the generation of HE cells with lymphoid potential from human pluripotent stem cells and for HE cell specification in mouse AGM^59,60^. Therefore, HE cells are essential for making blood cells in vitro^61^. HUVEC-E4 cells, which promote the reprogramming of adult mouse ECs into immunocompetent HSCs, can also transform aHE cells into iHSPCs, including aHE-iHSPC1 and aHE-iHSPC2. Recently, it has been reported that HSC-competent HE cells generate HSCs with multi-lineage differentiation and self-renewal capabilities, whereas non-HSC-competent HE cells produce phenotypic HPCs lacking significant multilineage engraftment ability^62^. This phenomenon is similar to our observation that aHE-iHSPC2 and aHE-iHSPC1 we identified here showed distinct properties, implying that the HE cells we used were a mixture containing both HSC-competent and non-HSC-competent HE cells. Moreover, the pHE cells, which also have arterial properties, give rise to pHE-iHSPCs with limited self-renewal ability in vitro. However, whether the pHE population contains HSC-competent HE cells needs to be further investigated. In addition, whether ECs from the mouse placenta after parturition have hematopoietic potential is unclear. Further study will focus on the molecular features of ECs and/or HE cells in the placenta at different stages, which would help reassess the medical value of the placenta after parturition.

According to previous reports, many genes and signals play roles in the development of HSCs. For example, *Runx1* has been widely reported to be essential for the EHT process and also for the development of HSCs^63–65^. Notch signaling is expressed in the AGM region and promotes HE specification and the emergence of HSCs^66–68^. Inflammatory signals also play a role in the emergence of HSCs in the AGM region^43,44,47,69^. Among them, Notch and inflammatory signals have also been shown to play a role in HSC induction in vitro^49,70^. In this study, we found that Notch signaling-related genes, *JAG1* and *HES4* and cytokines, IL1α and IL33, were highly expressed in HUVEC-E4 cells, which all have been reported to play roles in HE cell transformation^48,49,71^. In addition, we found that when blocking the function of CX3CL1 and IL1A, the generation of hematopoietic cells was impaired. Therefore, genes highly expressed in human HUVEC-E4 cells can promote the production of HSPCs from mouse HE cells. This raises a series of interesting questions, i.e., why human HUVECs secrete signals to promote the transformation of HE cells in mice. Is this due to the high homology of these molecular signals between humans and mice? Conversely, can mouse-derived cytokines promote the induction of human blood cells? Further investigation is warranted to address these issues.

In summary, our findings demonstrate that, HE cells from the AGM region and placenta, can be converted into aHE-iHSPC and pHE-iHSPC, respectively. However, the self-renewal ability of pHE-iHSPCs is limited. Furthermore, we identified that the self-renewal-associated genes are insufficiently expressed in pHE-iHSPC, and that RA treatment enhances the self-renewal ability of pHE-iHSPC. These results will support future application of pHE cells for hematopoietic cell generation for clinic use.

## Materials and methods

### Experiment models

Wild-type C57BL/6 mice were purchased from SPF (Beijing) Biotechnology Co.; B6-G/R mice (Strain NO. T006163) were purchased from GemPharmatech (Nanjing, China). The mice were used for timed mating, with the morning of detection of the vaginal plug defined as embryonic day (E) 0. For flow cytometry analysis, male B6-G/R mice were crossed with female C57BL/6 mice to obtain the AGM tissue and placenta, so, cells of the AGM region and the fetal component of placenta are GFP-positive. For the transplantation assay, GFP-positive AGM and placenta were used as donors for HE cells, and 8–12-week-old male C57BL/6 mice were used as recipients. The present study was approved by the Ethical Review Committee of the Institute of Zoology, Chinese Academy of Sciences, China.

The human umbilical cord was collected by Xiaokui Yang from the Beijing Obstetrics and Gynecology Hospital, Capital Medical University, for the isolation of HUVECs. The present study was approved by the Ethical Review Committee of the Beijing Obstetrics and Gynecology Hospital, Capital Medical University, Beijing Maternal and Child Health Care Hospital under the research license (2022-KY-074-01). Each pregnant woman involved in the project has signed the informed consent. All pregnant women in our research project share the same ethical approval license number.

### Immunofluorescence

Immunofluorescence assays for embryos and placentas were performed as previously reported^25,72^. Embryos were separated from pregnant mice and fixed in 4% paraformaldehyde (PFA)/PBS for 8–12 h at 4 °C, followed by 30% sucrose/PBS solution overnight. Placentas were separated from maternal decidua and umbilical cord, and then fixed in 1% PFA/PBS for 8–12 h at 4 °C, followed by 30% sucrose/PBS solution overnight. Then they were embedded and frozen in 100% OCT. Frozen sections were cut into 10-µm slices and blocked in 5% BSA (0.3% Triton X-100) for 1 h at room temperature, followed by incubation with anti-CD44 antibody (Abcam, Cat.# ab157107) and anti-Tie2 antibody (R&D systems, Cat.# AF762) diluted in 1% BSA overnight at 4 °C. After being washed, slides were incubated with the secondary antibody specific to Goat or Rabbit based on the resource of the primary antibody for 1 h at room temperature, and Hoechst was used to stain the nucleus. Immunofluorescent images were acquired by a confocal laser microscope (A1; Nikon) and processed using Nikon confocal software.

### Preparation of single cell suspensions

For the AGM region, tissues were washed in PBS twice on ice and transferred to pre-warmed digestion medium containing 0.1 g/mL collagenase (Sigma, Cat.# C2674) and 0.04% DNase (Sigma-Aldrich, Cat.# DN25). Tissues were gently pipetted and further incubated at 37 °C for 15–20 min with general shaking every 5 min. For placentas, tissues of pregnant mice were dissected and the maternal decidua and umbilical cord were removed. Tissues were washed in PBS twice on ice and transferred to pre-warmed digestion medium containing 0.1 g/mL collagenase and 0.04% DNase, and subsequently passed through 20G and 25G needles. Tissues were shaken for 30 s and further incubated at 37 °C for 30 min with general shaking every 10 min. Then tissues were centrifuged and washed in PBS and transferred to cell dissociation reagent (Gibco, Cat.# A1110501) for 6–8 min at 37 °C. For all tissues, cell suspensions were passed through 70 µm cell strainers (BD Falcon, Cat.# 352235) to obtain single cell suspensions.

### Flow cytometry analysis

Cells were stained with specific antibodies in FACS buffer (1× PBS with 2% fetal bovine serum (FBS)) for 30 min at 4 °C. Antibodies used in this study including anti-CD45 (eBioscience, Cat.# 48-0451-82), anti-CD45 (Biolegend, Cat.# 103110), anti-c-Kit (eBioscience, Cat.# 47-1171-82), anti-Ter119 (Biolegend, Cat.# 116210), anti-CD31 (eBioscience, Cat.# 25-0311-82), anti-CD44 (eBioscience, Cat.# 48-0441-82), anti-Ki67 (Biolegend, Cat.#350503), anti-CD41 (eBioscience, Cat.# 11-0411-81), anti-CD63 (Biolegend, Cat.# 143903) and anti-CD27 (eBioscience, Cat.# 17-0271-81). Live cells were separated with 7-AAD (Biolegend, Cat.# 420404). Cells were analyzed and sorted by BD FACS Aria Fusion. Data were further analyzed with FlowJo software.

### Preparation of HUVECs

HUVECs were obtained from the human umbilical cord and isolated by cell surface markers CD31 (Biolegend, Cat.# 303117) and CD144 (eBioscience, Cat.# 12-1449-80) using FACS. Then HUVECs were maintained in endothelial cell medium (ECM, Sciencell, Cat.# 1001) consisting of 5% FBS (Sciencell, Cat.# 0025), 1% endothelial cell growth supplement (Sciencell, Cat.# 1052) and 1% Penicillin-Streptomycin Solution (Sciencell, Cat.# 0503). These cells were maintained under standard cell culture conditions at 37 °C and 5% CO_2_.

### Lentivirus packaging and infection

The *E4ORF1* gene was amplified from the E4 plasmid (a gift from Dr. Bisen Ding) by PCR and subcloned into pCDH-CMV-MCS-EF1-copGFP lentiviral vector (purchased from Addgene). For lentivirus packaging, the shRNA constructs were transfected into 293T cells with pSPAX2 and pMD.2G, and the supernatant was collected after 48 h and filtered by 0.45 μm polyethersulfone membrane (Sartorius). Then lentivirus pellet was re-suspended in sterile ECM. For lentivirus infection, HUVECs were first cultured in a 24-well plate for 12 h before infection; then lentivirus was added into the cells along with HitransG P (Genechem, Cat.# REVG005) and incubated for 24 h.

### Cell survival assay

HUVEC-E4 cells and HUVECs were separately plated on 96-well plates at a density of 3 **×** 10^4^ per well. After 12 h culture in the ECM medium, the culture medium was replaced by serum/cytokine-free M199 (Day 0). The MTT-based alamarBlue (YEASEN, Cat.# 40202ES) was used to assess cell survival from Day 0 to Day 7.

### Hematopoietic cell induction and live-cell imaging

The AGM region and placentas were dissociated into single-cell suspensions before flow cytometry sorting. After antibody staining, 10,000 sorted HE cells (CD44^+^CD31^+^CD45^–^Ter119^–^7-AAD^–^) and non-HE cells (CD44^–^CD31^+^CD45^–^Ter119^–^7-AAD^–^) were co-cultured with HUVEC-E4 cells at 37 °C and 5% CO_2_ in StemSpan SFEM culture medium (STEMCELL Technologies, Cat.# 09650), 10% KnockOut Serum Replacement (Invitrogen, Cat.# 100110828028), 10 ng/mL hFGF-2 (bFGF, Peprotech, Cat.# 100-18), 50 ng/mL mouse c-Kit ligand (SCF, Peprotech, Cat.# 250-03). After 7 days, co-cultured cells were collected for further analysis. For time-lapse imaging, pHE cells and HUVEC-E4 cells were kept constantly in culture medium at 37 °C and 5% CO_2_ and imaged by Andor Dragonfly 505.

### CFU culture assay

For hematopoietic function experiments, the GFP^+^CD45^+^c-Kit^+^ iHPCs were harvested and cultured in MethoCult GF M3434 medium (Stem Cell Technologies, Cat.# 03434) in ultra-low attachment 24-well plates (Coring, Cat.# 3473). After cultured for 7–10 days at 37 °C and 5% CO_2_, the number of colonies including BFU-E, CFU-GM and CFU-GEMM was counted respectively. For HE potential experiments, HE cells (CD44^+^CD31^+^CD45^–^Ter119^–^7-AAD^–^) and hematopoietic cells (CD44^+^CD31^+^CD45^+^Ter119^–^7-AAD^–^) were harvested and subjected to CFU culture assay as above mentioned.

### Transplantation assay

C57BL/6 recipients received a split lethal dose (9 Gy) of X-ray irradiation (RS2000). For the primary transplantation assay, a total of 5000 GFP^+^CD45^+^c-Kit^+^ iHPCs were harvested from hematopoietic cell induction co-culture assay, and then mixed with 2 × 10^5^ nucleated BM cells from C57BL/6 mice. Finally, these cells were intravenously injected into the tail vein of the recipients.

### Quantitative real-time PCR

Total RNA from sorted cells was extracted using the QIAGEN RNeasy Mini Kit (Cat.# 74104) and reverse-transcribed with oligo-dT to generate cDNA for qPCR templates. The qPCR assays were performed with the Bio-Rad system, and the expression of mouse *Gapdh* was used as the internal control. For HUVEC cells, the expression of human *GAPDH* was used as the internal control. The sequences of the qPCR primers used are listed in Supplementary Table S1.

### Bulk RNA-seq analysis

Raw RNA-seq data from cell populations were first trimmed using Trim Galore to remove adapters and improve read quality (https://github.com/FelixKrueger/TrimGalore). Paired clean reads were mapped using hisat2 to a custom human reference genome that incorporates the E4ORF1 sequence. This custom genome was built with hisat2-build, using version GRCh38 from Ensembl as the reference^73^. Count matrix was calculated using HTSeq-count to count reads per gene based on the corresponding modified GTF annotation file^74^. The batch effect in the raw count table was corrected using the ComBat_seq function from the SVA R package^75^.

DESeq2 was used to normalize the count matrix and to identify DEGs with default parameters. *P*-value < 0.05 was set as the cutoff (Supplementary Table S2)^76^. GO term enrichment was calculated by R package clusterProfiler with function simplify (cutoff = 0.7) to remove redundant output from enrichGO function (Supplementary Table S2)^77^. ClusterProfiler was also used to conduct GSEA enrichment analysis using the gseGO function, while the visualization of the selected enriched results was performed with the gseaplot2 function (Supplementary Table S2)^77^.

### 10X genomics scRNA-seq data pre-processing

For 10X Genomics data, raw reads were mapped to custom reference built with mm10 reference genome to include zsgreen sequence using Cell Ranger software suite (version 6.1.2, https://github.com/10xGenomics/cellranger). Cells containing excessively high (> 50,000 counts, > 7500 features) or low (< 6000 counts, < 1000 features) counts and features were excluded; and a mitochondrial percentage cutoff of 0.1 was applied (Supplementary Table S3). For aHE induced cells, 3012 cells expressing GFP and Ptprc were retained for downstream analysis. After normalizing the data to a library size with a size factor of 10,000, dimension reduction was performed by UMAP using the top 30 principal components (PCs) obtained from the RunPCA function (Supplementary Table S3). We selected 0.3 as the resolution parameter for clustering using the Louvain algorithm with 20 PCs for local neighbor identification (Supplementary Table S3). For pHE induced cells, 2966 cells expressing GFP and Ptprc were retained for downstream analysis. After normalizing the data to a library size with a size factor of 10,000, dimension reduction was performed by UMAP using the top 30 PCs obtained from the RunPCA function (Supplementary Table S3). We selected 0.2 as the resolution parameter for clustering using Louvain algorithm with 20 PCs for local neighbor identification (Supplementary Table S3). Integration was performed using the FindIntegration Anchors function to identify anchors and the Integrate Data function afterward.

### DEG identification and GO enrichment analysis

We used the sc.tl.rank_genes_groups function in SCANPY to perform Wilcoxon rank-sum test to find DEGs among each cell cluster. Default parameters were used. Genes with *P*-value < 0.05 were thought as significant DEGs and shown on heatmap plots (Supplementary Tables S3–S5). GO term enrichment analysis was performed using the R package clusterProfiler, with the simplify function applied to remove redundant GO terms with a similarity higher than the cutoff (cutoff = 0.7). Top enriched GO terms (*P*-value < 0.05) were visualized using the enrichplot R package (Supplementary Tables S3–S5).

### Quantification and statistical analysis

GraphPad Prism 9 was used to analyze the data. The data are reported as the means ± SD. Unpaired two-tailed Student’s *t*-test was applied for statistical analyses.

## Supplementary information

Supplementary Information

Supplementary Table S1

Supplementary Table S2

Supplementary Table S3

Supplementary Table S4

Supplementary Table S5

Supplementary Video S1

## Data Availability

The raw sequence data reported in this paper have been deposited in the Genome Sequence Archive^78^ in the National Genomics Data Center^79^, China National Center for Bioinformation/Beijing Institute of Genomics, Chinese Academy of Sciences (GSA: *CRA011248* and *HRA004754*), and are publicly accessible at https://ngdc.cncb.ac.cn/gsa.

## References

[1] Rowe, R. G., Mandelbaum, J., Zon, L. I. & Daley, G. Q. Engineering hematopoietic stem cells: lessons from development. *Cell Stem Cell* **18**, 707–720 (2016). 27257760 10.1016/j.stem.2016.05.016 PMC4911194

[2] Zambidis, E. T., Peault, B., Park, T. S., Bunz, F. & Civin, C. I. Hematopoietic differentiation of human embryonic stem cells progresses through sequential hematoendothelial, primitive, and definitive stages resembling human yolk sac development. *Blood* **106**, 860–870 (2005). 15831705 10.1182/blood-2004-11-4522 PMC1895146

[3] Ebrahimi, M. et al. Differentiation of human induced pluripotent stem cells into erythroid cells. *Stem Cell Res. Ther.* **11**, 483 (2020). 10.1186/s13287-020-01998-9 PMC7667818 33198819

[4] Sugimura, R. et al. Haematopoietic stem and progenitor cells from human pluripotent stem cells. *Nature* **545**, 432–438 (2017). 28514439 10.1038/nature22370 PMC5872146

[5] Guo, R. et al. Guiding T lymphopoiesis from pluripotent stem cells by defined transcription factors. *Cell Res.* **30**, 21–33 (2020). 31729468 10.1038/s41422-019-0251-7 PMC6951346

[6] Amabile, G. et al. In vivo generation of transplantable human hematopoietic cells from induced pluripotent stem cells. *Blood* **121**, 1255–1264 (2013). 23212524 10.1182/blood-2012-06-434407 PMC3701251

[7] Piau, O. et al. Generation of transgene-free hematopoietic stem cells from human induced pluripotent stem cells. *Cell Stem Cell* **30**, 1610–1623.e7 (2023). 38065068 10.1016/j.stem.2023.11.002

[8] Sandler, V. M. et al. Reprogramming human endothelial cells to haematopoietic cells requires vascular induction. *Nature* **511**, 312–318 (2014). 25030167 10.1038/nature13547 PMC4159670

[9] Lis, R. et al. Conversion of adult endothelium to immunocompetent haematopoietic stem cells. *Nature* **545**, 439–445 (2017). 28514438 10.1038/nature22326 PMC5794215

[10] Barcia Duran, J. G., Lis, R., Lu, T. M. & Rafii, S. In vitro conversion of adult murine endothelial cells to hematopoietic stem cells. *Nat. Protoc.* **13**, 2758–2780 (2018). 30429596 10.1038/s41596-018-0060-3 PMC9923715

[11] Bertrand, J. Y. et al. Haematopoietic stem cells derive directly from aortic endothelium during development. *Nature* **464**, 108–111 (2010). 20154733 10.1038/nature08738 PMC2858358

[12] Boisset, J. C. et al. In vivo imaging of haematopoietic cells emerging from the mouse aortic endothelium. *Nature* **464**, 116–120 (2010). 20154729 10.1038/nature08764

[13] Chen, M. J. et al. Erythroid/myeloid progenitors and hematopoietic stem cells originate from distinct populations of endothelial cells. *Cell Stem Cell* **9**, 541–552 (2011). 22136929 10.1016/j.stem.2011.10.003 PMC3576591

[14] Gao, L. et al. RUNX1 and the endothelial origin of blood. *Exp. Hematol.* **68**, 2–9 (2018). 30391350 10.1016/j.exphem.2018.10.009 PMC6494457

[15] Frame, J. M., Fegan, K. H., Conway, S. J., McGrath, K. E. & Palis, J. Definitive hematopoiesis in the yolk sac emerges from Wnt-responsive hemogenic endothelium independently of circulation and arterial identity. *Stem Cells* **34**, 431–444 (2016). 26418893 10.1002/stem.2213 PMC4755868

[16] Kissa, K. & Herbomel, P. Blood stem cells emerge from aortic endothelium by a novel type of cell transition. *Nature* **464**, 112–115 (2010). 20154732 10.1038/nature08761

[17] Lee, L. K., Ueno, M., Van Handel, B. & Mikkola, H. K. Placenta as a newly identified source of hematopoietic stem cells. *Curr. Opin. Hematol.* **17**, 313–318 (2010). 20571394 10.1097/MOH.0b013e328339f295 PMC2929561

[18] Rhodes, K. E. et al. The emergence of hematopoietic stem cells is initiated in the placental vasculature in the absence of circulation. *Cell Stem Cell* **2**, 252–263 (2008). 18371450 10.1016/j.stem.2008.01.001 PMC2888040

[19] Mikkola, H. K., Gekas, C., Orkin, S. H. & Dieterlen-Lievre, F. Placenta as a site for hematopoietic stem cell development. *Exp. Hematol.* **33**, 1048–1054 (2005). 16140153 10.1016/j.exphem.2005.06.011

[20] Gekas, C., Dieterlen-Lievre, F., Orkin, S. H. & Mikkola, H. K. The placenta is a niche for hematopoietic stem cells. *Dev. Cell* **8**, 365–375 (2005). 15737932 10.1016/j.devcel.2004.12.016

[21] Palis, J. & Yoder, M. C. Yolk-sac hematopoiesis: the first blood cells of mouse and man. *Exp. Hematol.* **29**, 927–936 (2001). 11495698 10.1016/s0301-472x(01)00669-5

[22] Palis, J., Robertson, S., Kennedy, M., Wall, C. & Keller, G. Development of erythroid and myeloid progenitors in the yolk sac and embryo proper of the mouse. *Development* **126**, 5073–5084 (1999). 10529424 10.1242/dev.126.22.5073

[23] Zhao, Y. X. et al. Single-cell RNA sequencing-guided fate-mapping toolkit delineates the contribution of yolk sac erythro-myeloid progenitors. *Cell Rep.* **42**, 113364 (2023). 37922312 10.1016/j.celrep.2023.113364

[24] Lee, L. K. et al. LYVE1 marks the divergence of yolk sac definitive hemogenic endothelium from the primitive erythroid lineage. *Cell Rep.* **17**, 2286–2298 (2016). 27880904 10.1016/j.celrep.2016.10.080 PMC6940422

[25] Liang, G. et al. De novo generation of macrophage from placenta-derived hemogenic endothelium. *Dev. Cell* **56**, 2121–2133.e6 (2021). 34197725 10.1016/j.devcel.2021.06.005

[26] Li, Z. et al. Generation of hematopoietic stem cells from purified embryonic endothelial cells by a simple and efficient strategy. *J. Genet. Genomics* **40**, 557–563 (2013). 24238609 10.1016/j.jgg.2013.09.001

[27] Zhou, F. et al. Tracing haematopoietic stem cell formation at single-cell resolution. *Nature* **533**, 487–492 (2016). 27225119 10.1038/nature17997

[28] Zhou, J. et al. Combined single-cell profiling of lncrnas and functional screening reveals that h19 is pivotal for embryonic hematopoietic stem cell development. *Cell Stem Cell* **24**, 285–298.e5 (2019). 30639035 10.1016/j.stem.2018.11.023

[29] Rybtsov, S. et al. Hierarchical organization and early hematopoietic specification of the developing HSC lineage in the AGM region. *J. Exp. Med.* **208**, 1305–1315 (2011). 21624936 10.1084/jem.20102419 PMC3173253

[30] Taoudi, S. et al. Extensive hematopoietic stem cell generation in the AGM region via maturation of VE-cadherin+CD45+ pre-definitive HSCs. *Cell Stem Cell* **3**, 99–108 (2008). 18593562 10.1016/j.stem.2008.06.004

[31] Li, Y. Q. et al. Spatiotemporal and functional heterogeneity of hematopoietic stem cell-competent hemogenic endothelial cells in mouse embryos. *Front. Cell Dev. Biol.* **9**, 699263 (2021). 10.3389/fcell.2021.699263 PMC8385538 34458261

[32] Chanda, B., Ditadi, A., Iscove, N. N. & Keller, G. Retinoic acid signaling is essential for embryonic hematopoietic stem cell development. *Cell* **155**, 215–227 (2013). 24074870 10.1016/j.cell.2013.08.055

[33] Luff, S. A. et al. Identification of a retinoic acid-dependent haemogenic endothelial progenitor from human pluripotent stem cells. *Nat. Cell Biol.* **24**, 616–624 (2022). 35484246 10.1038/s41556-022-00898-9 PMC9109599

[34] Thambyrajah, R. et al. IkappaBalpha controls dormancy in hematopoietic stem cells via retinoic acid during embryonic development. *Nat. Commun.* **15**, 46735 (2024). 10.1038/s41467-024-48854-5 PMC11144194 38824124

[35] Fowler, J. L. et al. Lineage-tracing hematopoietic stem cell origins in vivo to efficiently make human HLF+ HOXA+ hematopoietic progenitors from pluripotent stem cells. *Dev. Cell* **59**, 1110–1131.e22 (2024). 38569552 10.1016/j.devcel.2024.03.003 PMC11072092

[36] Purton, L. E., Bernstein, I. D. & Collins, S. J. All-trans retinoic acid enhances the long-term repopulating activity of cultured hematopoietic stem cells. *Blood* **95**, 470–477 (2000). 10627451

[37] Purton, L. E., Bernstein, I. D. & Collins, S. J. All-trans retinoic acid delays the differentiation of primitive hematopoietic precursors (lin-c-kit+Sca-1(+)) while enhancing the terminal maturation of committed granulocyte/monocyte progenitors. *Blood* **94**, 483–495 (1999). 10397716

[38] Cabezas-Wallscheid, N. et al. Vitamin A-retinoic acid signaling regulates hematopoietic stem cell dormancy. *Cell* **169**, 807–823.e19 (2017). 28479188 10.1016/j.cell.2017.04.018

[39] Seandel, M. et al. Generation of a functional and durable vascular niche by the adenoviral E4ORF1 gene. *Proc. Natl. Acad. Sci. USA* **105**, 19288–19293 (2008). 10.1073/pnas.0805980105 PMC2588414 19036927

[40] Alvarez-Silva, M., Belo-Diabangouaya, P., Salaun, J. & Dieterlen-Lievre, F. Mouse placenta is a major hematopoietic organ. *Development* **130**, 5437–5444 (2003). 14507780 10.1242/dev.00755

[41] Oatley, M. et al. Single-cell transcriptomics identifies CD44 as a marker and regulator of endothelial to haematopoietic transition. *Nat. Commun.* **11**, 586 (2020). 31996681 10.1038/s41467-019-14171-5 PMC6989687

[42] Hou, S. et al. Embryonic endothelial evolution towards first hematopoietic stem cells revealed by single-cell transcriptomic and functional analyses. *Cell Res.* **30**, 376–392 (2020). 32203131 10.1038/s41422-020-0300-2 PMC7196075

[43] Espin-Palazon, R. et al. Proinflammatory signaling regulates hematopoietic stem cell emergence. *Cell* **159**, 1070–1085 (2014). 25416946 10.1016/j.cell.2014.10.031 PMC4243083

[44] He, Q. et al. Inflammatory signaling regulates hematopoietic stem and progenitor cell emergence in vertebrates. *Blood* **125**, 1098–1106 (2015). 25540193 10.1182/blood-2014-09-601542

[45] Capitano, M. L. Toll-like receptor signaling in hematopoietic stem and progenitor cells. *Curr. Opin. Hematol.* **26**, 207–213 (2019). 31033704 10.1097/MOH.0000000000000511

[46] Mariani, S. A. et al. Pro-inflammatory aorta-associated macrophages are involved in embryonic development of hematopoietic stem cells. *Immunity* **50**, 1439–1452 (2019). 31178352 10.1016/j.immuni.2019.05.003 PMC6591003

[47] Li, Y. et al. Inflammatory signaling regulates embryonic hematopoietic stem and progenitor cell production. *Genes Dev.* **28**, 2597–2612 (2014). 25395663 10.1101/gad.253302.114 PMC4248291

[48] Calvanese, V. et al. Mapping human haematopoietic stem cells from haemogenic endothelium to birth. *Nature* **604**, 534–540 (2022). 35418685 10.1038/s41586-022-04571-x PMC9645817

[49] Zhang, Y., Kang, Z., Liu, M., Wang, L. & Liu, F. Single-cell omics identifies inflammatory signaling as a trans-differentiation trigger in mouse embryos. *Dev. Cell* **59**, 961–978.e7 (2024). 38508181 10.1016/j.devcel.2024.02.010

[50] Gekas, C. et al. Hematopoietic stem cell development in the placenta. *Int. J. Dev. Biol.* **54**, 1089–1098 (2010). 20711986 10.1387/ijdb.103070cg PMC4285582

[51] Hamey, F. K. & Gottgens, B. Machine learning predicts putative hematopoietic stem cells within large single-cell transcriptomics data sets. *Exp. Hematol.* **78**, 11–20 (2019). 31513832 10.1016/j.exphem.2019.08.009 PMC6900257

[52] Zhu, Q. et al. Developmental trajectory of prehematopoietic stem cell formation from endothelium. *Blood* **136**, 845–856 (2020). 32392346 10.1182/blood.2020004801 PMC7426642

[53] Florez, M. A. et al. Interferon gamma mediates mematopoietic stem cell activation and niche relocalization through BST2. *Cell Rep.* **33**, 108530 (2020). 33357430 10.1016/j.celrep.2020.108530 PMC7816211

[54] Kitajima, K., Shingai, M., Ando, H., Hamasaki, M. & Hara, T. An interferon-gamma/FLT3 axis positively regulates hematopoietic progenitor cell expansion from human pluripotent stem cells. *Stem Cells* **40**, 906–918 (2022). 35901509 10.1093/stmcls/sxac052

[55] Tomellini, E. et al. Integrin-alpha3 is a functional marker of ex vivo expanded human long-term hematopoietic stem cells. *Cell Rep.* **28**, 1063–1073.e5 (2019). 31340144 10.1016/j.celrep.2019.06.084

[56] Li, Y., Gao, L., Hadland, B., Tan, K. & Speck, N. A. CD27 marks murine embryonic hematopoietic stem cells and type II prehematopoietic stem cells. *Blood* **130**, 372–376 (2017). 28588017 10.1182/blood-2017-03-776849 PMC5520475

[57] Hu, M. et al. CD63 acts as a functional marker in maintaining hematopoietic stem cell quiescence through supporting TGFbeta signaling in mice. *Cell Death Differ* **29**, 178–191 (2022). 34363017 10.1038/s41418-021-00848-2 PMC8738745

[58] Cumano, A., Dieterlen-Lievre, F. & Godin, I. Lymphoid potential, probed before circulation in mouse, is restricted to caudal intraembryonic splanchnopleura. *Cell* **86**, 907–916 (1996). 8808626 10.1016/s0092-8674(00)80166-x

[59] Slukvin, I. I. & Uenishi, G. I. Arterial identity of hemogenic endothelium: a key to unlock definitive hematopoietic commitment in human pluripotent stem cell cultures. *Exp. Hematol.* **71**, 3–12 (2019). 30500414 10.1016/j.exphem.2018.11.007 PMC6401300

[60] Park, M. A. et al. Activation of the arterial program drives development of definitive hemogenic endothelium with lymphoid potential. *Cell Rep.* **23**, 2467–2481 (2018). 29791856 10.1016/j.celrep.2018.04.092 PMC6410360

[61] Blaser, B. W. & Zon, L. I. Making HSCs in vitro: don’t forget the hemogenic endothelium. *Blood* **132**, 1372–1378 (2018). 30089629 10.1182/blood-2018-04-784140 PMC6161767

[62] Dignum, T. et al. Multipotent progenitors and hematopoietic stem cells arise independently from hemogenic endothelium in the mouse embryo. *Cell Rep.* **36**, 109675 (2021). 34525376 10.1016/j.celrep.2021.109675 PMC8478150

[63] Chen, M. J., Yokomizo, T., Zeigler, B. M., Dzierzak, E. & Speck, N. A. Runx1 is required for the endothelial to haematopoietic cell transition but not thereafter. *Nature* **457**, 887–891 (2009). 19129762 10.1038/nature07619 PMC2744041

[64] Mukouyama, Y. et al. The AML1 transcription factor functions to develop and maintain hematogenic precursor cells in the embryonic aorta-gonad-mesonephros region. *Dev. Biol.* **220**, 27–36 (2000). 10720428 10.1006/dbio.2000.9617

[65] Wang, Q. et al. The CBFbeta subunit is essential for CBFalpha2 (AML1) function in vivo. *Cell* **87**, 697–708 (1996). 8929538 10.1016/s0092-8674(00)81389-6

[66] Zhang, C. et al. m(6)A modulates haematopoietic stem and progenitor cell specification. *Nature* **549**, 273–276 (2017). 28869969 10.1038/nature23883

[67] Lizama, C. O. et al. Repression of arterial genes in hemogenic endothelium is sufficient for haematopoietic fate acquisition. *Nat. Commun.* **6**, 7739 (2015). 26204127 10.1038/ncomms8739 PMC4519987

[68] Gama-Norton, L. et al. Notch signal strength controls cell fate in the haemogenic endothelium. *Nat. Commun.* **6**, 8510 (2015). 26465397 10.1038/ncomms9510 PMC4634136

[69] Espin-Palazon, R., Weijts, B., Mulero, V. & Traver, D. Proinflammatory signals as fuel for the fire of hematopoietic stem cell emergence. *Trends Cell Biol.* **28**, 58–66 (2018). 28882414 10.1016/j.tcb.2017.08.003

[70] Hadland, B. K. et al. Endothelium and NOTCH specify and amplify aorta-gonad-mesonephros-derived hematopoietic stem cells. *J. Clin. Invest.* **125**, 2032–2045 (2015). 25866967 10.1172/JCI80137 PMC4463208

[71] Orelio, C., Haak, E., Peeters, M. & Dzierzak, E. Interleukin-1-mediated hematopoietic cell regulation in the aorta-gonad-mesonephros region of the mouse embryo. *Blood* **112**, 4895–4904 (2008). 18805969 10.1182/blood-2007-12-123836 PMC2597597

[72] Lv, J., Wang, L., Gao, Y., Ding, Y. Q. & Liu, F. 5-hydroxytryptamine synthesized in the aorta-gonad-mesonephros regulates hematopoietic stem and progenitor cell survival. *J. Exp. Med.* **214**, 529–545 (2017). 28031476 10.1084/jem.20150906 PMC5294845

[73] Kim, D., Paggi, J. M., Park, C., Bennett, C. & Salzberg, S. L. Graph-based genome alignment and genotyping with HISAT2 and HISAT-genotype. *Nat. Biotechnol.* **37**, 907–915 (2019). 31375807 10.1038/s41587-019-0201-4 PMC7605509

[74] Putri, G. H., Anders, S., Pyl, P. T., Pimanda, J. E. & Zanini, F. Analysing high-throughput sequencing data in Python with HTSeq 2.0. *Bioinformatics* **38**, 2943–2945 (2022). 35561197 10.1093/bioinformatics/btac166 PMC9113351

[75] Leek, J. T., Johnson, W. E., Parker, H. S., Jaffe, A. E. & Storey, J. D. The sva package for removing batch effects and other unwanted variation in high-throughput experiments. *Bioinformatics* **28**, 882–883 (2012). 22257669 10.1093/bioinformatics/bts034 PMC3307112

[76] Love, M. I., Huber, W. & Anders, S. Moderated estimation of fold change and dispersion for RNA-seq data with DESeq2. *Genome Biol.* **15**, 1–21 (2014). 10.1186/s13059-014-0550-8 PMC4302049 25516281

[77] Yu, G., Wang, L.-G., Han, Y. & He, Q.-Y. clusterProfiler: an R package for comparing biological themes among gene clusters. *Omics J. Integr. Biol.* **16**, 284–287 (2012). 10.1089/omi.2011.0118 PMC3339379 22455463

[78] Chen, T. et al. The genome sequence archive family: toward explosive data growth and diverse data types. *Genomics Proteom. Bioinform.* **19**, 578–583 (2021). 10.1016/j.gpb.2021.08.001 PMC9039563 34400360

[79] Members, C.-N. Database resources of the national genomics data center, China national center for bioinformation in 2022. *Nucleic Acids Res.* **50**, D27–D38 (2022). 34718731 10.1093/nar/gkab951 PMC8728233

