# Supplementary material for: Conversion of placental hemogenic endothelial cells to hematopoietic stem and progenitor cells
Source: Cell Discov. 2025 Jan 28;11:9. doi: 10.1038/s41421-024-00760-2 (PMC11775181; doi:10.1038/s41421-024-00760-2)
Supplement: Supplementary file 1 — Supplementary Information [file 41421_2024_760_MOESM1_ESM.pdf]

Fig. S1

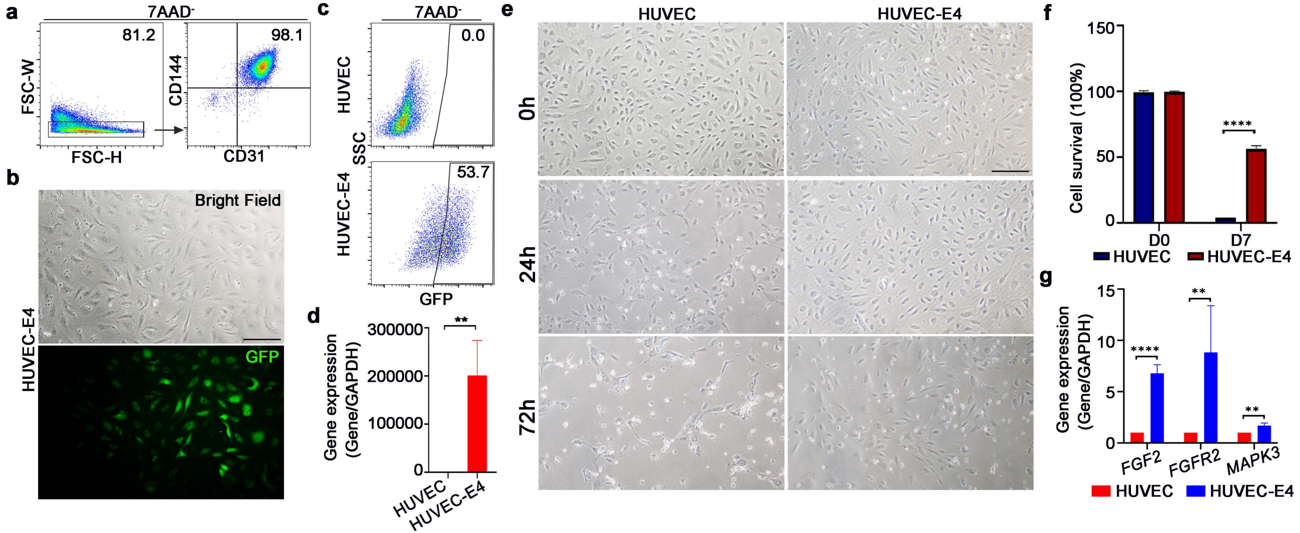
a
7AAD-
81.2
98.1
FSC-W
FSC-H
CD144
CD31
b
Bright Field
GFP
HUVEC-E4
c
7AAD-
0.0
53.7
HUVEC
HUVEC-E4
SSC
GFP
d
Gene expression (Gene/GAPDH)
300000
200000
100000
0
**
HUVEC
HUVEC-E4
e
HUVEC
HUVEC-E4
0h
24h
72h
f
Cell survival (100%)
150
100
50
0
****
D0
D7
HUVEC
HUVEC-E4
g
Gene expression (Gene/GAPDH)
15
10
5
0
****
**
**
FGF2
FGFR2
MAPK3
HUVEC
HUVEC-E4

Fig. S1 Establishment of vascular niche endothelial cells. (**a**) Flow cytometry analysis showing the expression of CD31 and CD144 of human umbilical vein endothelial cells (HUVEC). (**b**) Microscopy results showing the GFP expression in HUVEC-E4 cells transfected with the adenoviral E4ORF1 gene. (**c**) Flow cytometry analysis showing the expression of GFP in HUVEC-E4 cells transfected with the adenoviral E4ORF1 gene. (**d**) qPCR analysis showing the expression of E4ORF1 in HUVEC and HUVEC-E4 cells, respectively, n=3. (**e**) Microscopy results showing the survival of primary ECs with or without E4ORF1 expression. (**f**) Statistics results of the survival of primary ECs with or without E4ORF1 expression. (**g**) qPCR analysis showing the expression of *FGF2*, *FGFR2* and *MAPK3* in HUVEC and HUVEC-E4 cells, respectively, n=3. Scale bars, 50 μm. Error bars, mean ± s.d. Asterisk presents statistical significance (**$P < 0.01$, **** $P < 0.0001$). P values were calculated by two-tailed unpaired Student's t-test.

Fig.S2

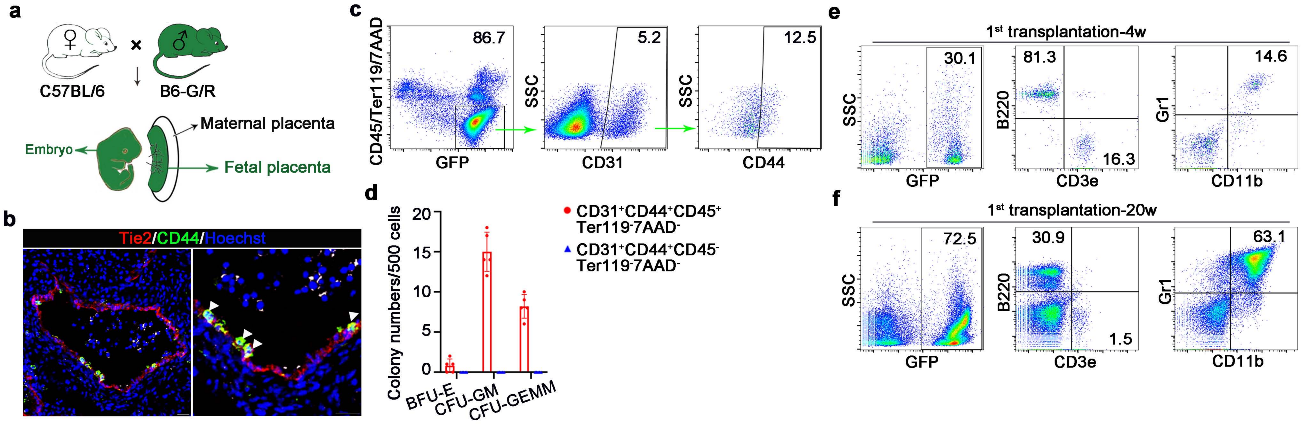

a
C57BL/6
B6-G/R
Maternal placenta
Embryo
Fetal placenta
b
Tie2/CD44/Hoechst
c
CD45/Ter119/7AAD
GFP
86.7
SSC
CD31
5.2
SSC
CD44
12.5
d
Colony numbers/500 cells
20
15
10
5
0
BFU-E
CFU-GM
CFU-GEMM
CD31+CD44+CD45+ Ter119-7AAD-
CD31+CD44+CD45- Ter119-7AAD-
e
1st transplantation-4w
SSC
GFP
30.1
B220
CD3e
81.3
16.3
Gr1
CD11b
14.6
f
1st transplantation-20w
SSC
GFP
72.5
B220
CD3e
30.9
1.5
Gr1
CD11b
63.1

Fig. S2 Functional analysis of aHE cells and aHE-iHPCs. (**a**) Schematic illustration of mating strategies for obtaining endothelial cells derived from embryos. (**b**) Immunofluorescence (IF) analysis of AGM region showing the co-localization of Tie2 and CD44 staining at E11.0, double positive cells are marked with white arrows. (**c**) Representative FACS plots showing the proportion of $CD44^+$ ECs in AGM region. (**d**) CFU-C assay of aHE cells ($CD31^+CD44^+CD45^-Ter119^-7AAD^-$) and hematopoietic cells ($CD31^+CD44^+CD45^+Ter119^-7AAD^-$), respectively. n=3. (**e**) Representative flow cytometry analysis showing the short term (peripheral blood, 4 weeks) repopulation of primary recipient transplanted with aHE-iHSPC. (**f**) Representative flow cytometry analysis showing the long-term (bone marrow, 20 weeks) repopulation of primary recipient transplanted with aHE-iHPCs. Scale bars, 50 μm. Error bars, mean ± s.d.

Fig. S3

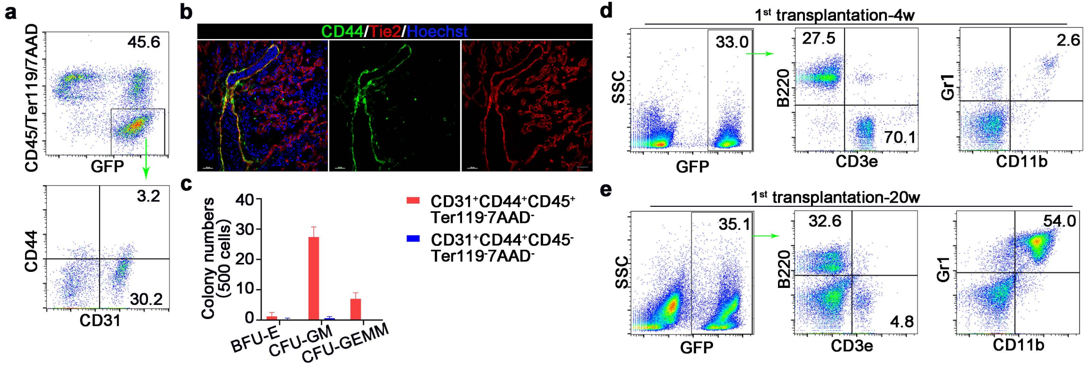
a
CD45/Ter119/7AAD
GFP
45.6
CD44
CD31
3.2
30.2
b
CD44/Tie2/Hoechst
c
Colony numbers (500 cells)
40
30
20
10
0
BFU-E
CFU-GM
CFU-GEMM
CD31+CD44+CD45+ Ter119-7AAD-
CD31+CD44+CD45- Ter119-7AAD-
d
1st transplantation-4w
SSC
GFP
33.0
B220
CD3e
27.5
70.1
Gr1
CD11b
2.6
e
1st transplantation-20w
SSC
GFP
35.1
B220
CD3e
32.6
4.8
Gr1
CD11b
54.0

Fig. S3 Functional analysis of pHE cells and pHE-iHPCs. (**a**) Representative FACS plots showing the proportion of $CD44^+$ ECs in the placenta. (**b**) IF analysis of placenta showing the co-localization of Tie2 and CD44 staining at E11.0, double positive cells are marked with white arrows. (**c**) CFU-C assay of pHE cells ($CD31^+CD44^+CD45^-Ter119^-7AAD^-$) and hematopoietic cells ($CD31^+CD44^+CD45^+Ter119^-7AAD^-$) from the placenta, respectively, n=3. (**d**) Representative flow cytometry analysis showing the short term (peripheral blood, 4 weeks) repopulation of primary recipient transplanted with pHE-iHPCs. (**e**) Representative flow cytometry analysis showing the long-term (bone marrow, 20 weeks) repopulation of primary recipient transplanted with pHE-iHSPC. Scale bars, 50 μm.

Fig. S4

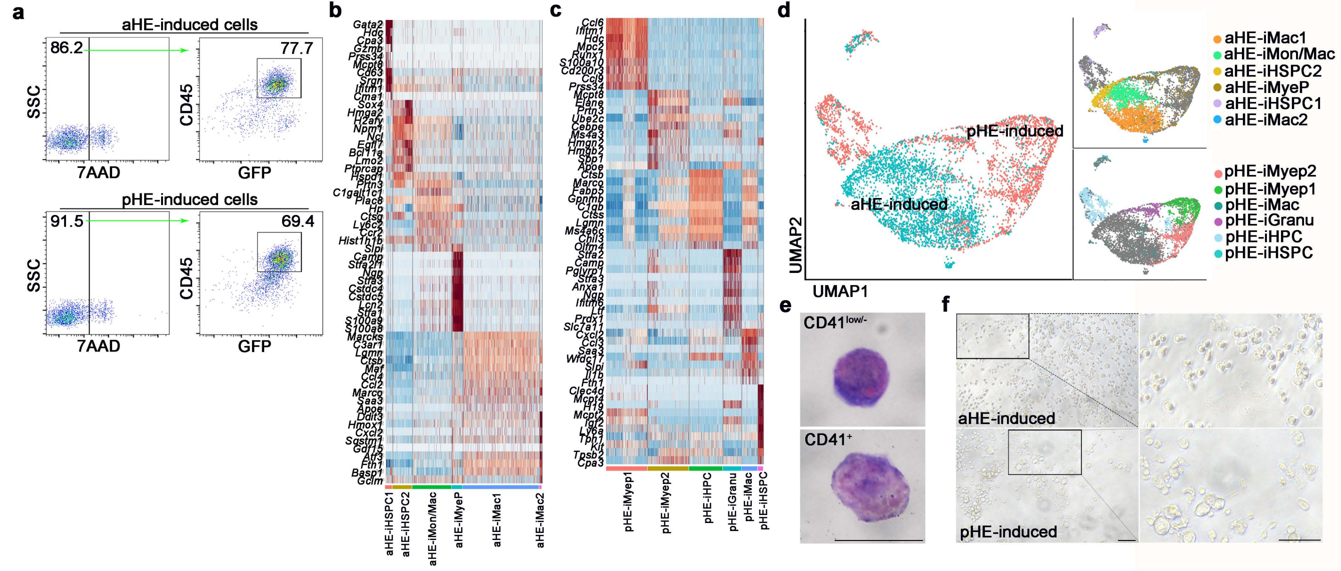
a
aHE-induced cells
86.2
77.7
SSC
7AAD
CD45
GFP
pHE-induced cells
91.5
69.4
SSC
7AAD
CD45
GFP
b
Gata2 Hdc Cpa3 Gzmb Prss34 Mcpt8 Cd63 Srgn Ifitm1 Cma1 Sox4 Hmga2 H2afy Npm1 Ncl Egfl7 Bcl11a Lmo2 Ptprcap Hspd1 Prtn3 C1galt1c1 Plac8 Hp Ctsg Ly6c2 Ccr2 Hist1h1b Slpi Camp Stfa2l1 Ngp Stfa3 Cstdc4 Cstdc5 Lcn2 Stfa1 S100a9 S100a8 Marcks C3ar1 Lgmn Ctsb Maf Ccl4 Ccl2 Marco Saa3 Apoe Ddit3 Hmox1 Cxcl2 Sqstm1 Gdf15 Atf3 Fth1 Basp1 Gclm
aHE-iHSPC1
aHE-iHSPC2
aHE-iMon/Mac
aHE-iMyeP
aHE-iMac1
aHE-iMac2
c
Ccl6 Ifitm1 Hdc Mpc2 Runx1 S100a10 Cd200r3 Ccl9 Prss34 Mcpt8 Elane Prtn3 Ube2c Cebpe Ms4a3 Hmgn2 Hmgb2 Sbp1 Apoe Ctsb Marco Fabp5 Gpnmb C1qb Ctss Lgmn Ms4a6c Chil3 Olfm4 Stfa2 Camp Pglyrp1 Stfa3 Anxa1 Ngp Ifitm6 Ltf Prdx1 Slc7a11 Cxcl2 Ccl3 Saa3 Wfdc17 Slpi Il1b Fth1 Clec4d Mcpt4 H19 Mcpt2 Igf2 Ly6a Tph1 Kit Tpsb2 Cpa3
pHE-iMyep1
pHE-iMyep2
pHE-iHPC
pHE-iGranu
pHE-iMac
pHE-iHSPC
d
pHE-induced
aHE-induced
UMAP2
UMAP1
aHE-iMac1
aHE-iMon/Mac
aHE-iHSPC2
aHE-iMyeP
aHE-iHSPC1
aHE-iMac2
pHE-iMyep2
pHE-iMyep1
pHE-iMac
pHE-iGranu
pHE-iHPC
pHE-iHSPC
e
CD41low/-
CD41+
f
aHE-induced
pHE-induced

Fig. S4 Collection and scRNA-seq analysis of aHE-iHCs and pHE-iHCs. (**a**) Representative FACS plots showing the proportions of GFP cells after AGM and placental HE cells co-cultured with HUVEC-E4 for 7 days. (**b**) Heatmap showing the DEGs for GFP^{+} aHE-iHC clusters. (**c**) Heatmap showing the DEGs for GFP^{+} pHE-iHC clusters. (**d**) Cell clusters in scRNA-seq data visualized by UMAP. (**e**) Giemsa staining showed the morphology of CD41$^{low/-}$ and CD41^{+} cells. Scale bars, 10 μm. (**f**) Morphology of cells after induced by aHE and pHE cells. Among them, only pHE cells produced significantly large round cells after induction. Scale bars, 50 μm.

Fig. S5

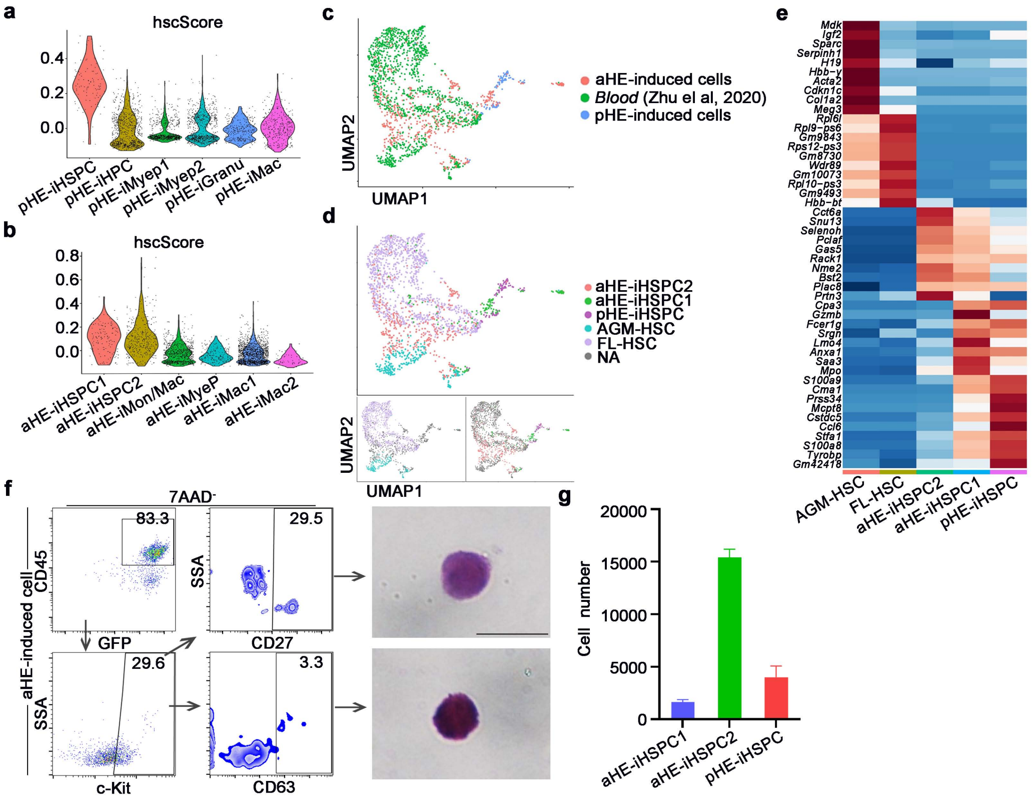

a
hscScore
0.4
0.2
0.0
pHE-iHSPC
pHE-iHPC
pHE-iMyep1
pHE-iMyep2
pHE-iGranu
pHE-iMac
b
hscScore
0.8
0.6
0.4
0.2
0.0
aHE-iHSPC1
aHE-iHSPC2
aHE-iMon/Mac
aHE-iMyeP
aHE-iMac1
aHE-iMac2
c
UMAP2
UMAP1
aHE-induced cells
Blood (Zhu el al, 2020)
pHE-induced cells
d
aHE-iHSPC2
aHE-iHSPC1
pHE-iHSPC
AGM-HSC
FL-HSC
NA
UMAP2
UMAP1
e
Mdk
Igf2
Sparc
Serpinh1
H19
Hbb-y
Acta2
Cdkn1c
Col1a2
Meg3
Rpl6l
Rpl9-ps6
Gm9843
Rps12-ps3
Gm8730
Wdr89
Gm10073
Rpl10-ps3
Gm9493
Hbb-bt
Cct6a
Snu13
Selenoh
Pclaf
Gas5
Rack1
Nme2
Bst2
Plac8
Prtn3
Cpa3
Gzmb
Fcer1g
Srgn
Lmo4
Anxa1
Saa3
Mpo
S100a9
Cma1
Prss34
Mcpt8
Cstdc5
Ccl6
Stfa1
S100a8
Tyrobp
Gm42418
AGM-HSC
FL-HSC
aHE-iHSPC2
aHE-iHSPC1
pHE-iHSPC
f
7AAD-
aHE-induced cell
CD45
GFP
83.3
SSA
c-Kit
29.6
SSA
CD27
29.5
CD63
3.3
g
20000
15000
10000
5000
0
Cell number
aHE-iHSPC1
aHE-iHSPC2
pHE-iHSPC

Fig. S5 Descript of the features of iHSPC populations. (**a**) Violin plot showing the hscScore analysis of pHE-iHC population. (**b**) Violin plot showing the hscScore analysis of aHE-iHC population. (**c**) UMAP showing the integration of iHSPC clusters from our data and HSC clusters from previous published data (Zhu et al., 2020). (**d**) UMAP demonstrated the integration of aHE-iHSPCs and pHE-iHSPCs with HSCs. (**e**) Heatmap showing the DEGs in AGM-HSC, FL-HSC, aHE-iHSPC2, aHE-iHSPC1 and pHE-iHSPC clusters. (**f**) Representative FACS plots show two groups of aHE-iHSPCs characterized by CD27 and CD63 in aHE-iHPCs (left), and Giemsa staining shows the morphology of $CD27^+$ aHE-iHSPCs and $CD63^+$ aHE-iHSPCs (right). Scale bars, 10 μm. (**g**) Bar plot showing the cell number of aHE-iHSPC1 and aHE-iHSPC2 after aHE cells co-cultured with HUVEC-E4 for 7 days and also pHE-iHSPC after pHE cells co-cultured with HUVEC-E4 for 7 days.

Supplementary Tables

Supplementary Table S1 qPCR primers used in this study

Supplementary Table S2 Information of Bulk RNA sequencing data

The sample information, DEGs of HUVEC(Ctrl) and HUVEC-E4 (E4) and GO terms enriched in HUVEC(Ctrl) and HUVEC-E4 (E4).

Supplementary Table S3 Information of 10× Genomics Single Cells RNA Sequencing Data

The sample information, cell annotation of aHE-induced and pHE-induced cells.

Supplementary Table S4 Information of integrated data

The sample used in the analysis of integrated data, DEGs of AGM-HSC, FL-HSC, aHE-iHSPC1, aHE-iHSPC2 and pHE-iHSPC and GO terms enriched in AGM-HSC, FL-HSC, aHE-iHSPC1, aHE-iHSPC2 and pHE-iHSPC.

Supplementary Table S5 Information of iHSPC

DEGs of aHE-iHSPC1, aHE-iHSPC2 and pHE-iHSPC.

Supplementary Video

Supplementary Video S1 Time-lapse imaging of endothelial-to-hematopoietic transition of pHE cells co-culture on HUVEC-E4.
